# Deciphering glial contributions to CSF1R-related disorder via single-nuclear transcriptomic profiling: a case study

**DOI:** 10.1186/s40478-024-01853-5

**Published:** 2024-08-28

**Authors:** Jie Pan, Jaume Fores-Martos, Claire Delpirou Nouh, Tanner D. Jensen, Kristen Vallejo, Romain Cayrol, Saman Ahmadian, Euan A. Ashley, Michael D. Greicius, Inma Cobos

**Affiliations:** 1 Department of Pathology, Stanford University School of Medicine, Stanford, CA USA; 2 Department of Neurology and Neurological Sciences, Stanford University School of Medicine, Stanford, CA USA; 3 Department of Pathology and Cell Biology, Faculty of Medicine, Université de Montréal, Montreal, QC Canada; 4 Department of Pathology, The Ohio State University Wexner Medical Center, Columbus, OH USA; 5 Department of Medicine, Stanford University School of Medicine, Stanford, CA USA; 6 Department of Genetics, Stanford University School of Medicine, Stanford, CA USA

**Keywords:** ALSP, HDLS, CSF1R, Microglia, Macrophage, Oligodendrocyte, OPC, GPNMB, Leukodystrophy

## Abstract

CSF1R-related disorder (CSF1R-RD) is a neurodegenerative condition that predominantly affects white matter due to genetic alterations in the *CSF1R* gene, which is expressed by microglia. We studied an elderly man with a hereditary, progressive dementing disorder of unclear etiology. Standard genetic testing for leukodystrophy and other neurodegenerative conditions was negative. Brain autopsy revealed classic features of adult-onset leukoencephalopathy with axonal spheroids and pigmented glia (ALSP), including confluent white matter degeneration with axonal spheroids and pigmented glial cells in the affected white matter, consistent with CSF1R-RD. Subsequent long-read sequencing identified a novel deletion in *CSF1R* that was not detectable with short-read exome sequencing. To gain insight into potential mechanisms underlying white matter degeneration in CSF1R-RD, we studied multiple brain regions exhibiting varying degrees of white matter pathology. We found decreased CSF1R transcript and protein across brain regions, including intact white matter. Single nuclear RNA sequencing (snRNAseq) identified two disease-associated microglial cell states: lipid-laden microglia (expressing *GPNMB*, *ATG7*, *LGALS1*, *LGALS3*) and inflammatory microglia (expressing *IL2RA*, *ATP2C1*, *FCGBP*, *VSIR*, *SESN3*), along with a small population of *CD44*^+^ peripheral monocyte-derived macrophages exhibiting migratory and phagocytic signatures. GPNMB^+^ lipid-laden microglia with ameboid morphology represented the end-stage disease microglia state. Disease-associated oligodendrocytes exhibited cell stress signatures and dysregulated apoptosis-related genes. Disease-associated oligodendrocyte precursor cells (OPCs) displayed a failure in their differentiation into mature myelin-forming oligodendrocytes, as evidenced by upregulated *LRP1, PDGFRA, SOX5, NFIA*, and downregulated *NKX2-2, NKX6.2, SOX4, SOX8, TCF7L2, YY1, ZNF488*. Overall, our findings highlight microglia–oligodendroglia crosstalk in demyelination, with CSF1R dysfunction promoting phagocytic and inflammatory microglia states, an arrest in OPC differentiation, and oligodendrocyte depletion.

**Supplementary Information:**

The online version contains supplementary material available at https://doi.org/10.1186/s40478-024-01853-5.

## Introduction

CSF1R-RD is an autosomal dominant neurodegenerative disorder of the white matter caused by mutations in the colony-stimulating factor-1 receptor (*CSF1R*) [14, 35, 62], a gene expressed in microglia. As such, it represents a subtype of primary microgliopathies [75]. The condition has been previously known by the descriptive neuropathological term ‘adult-onset leukoencephalopathy with axonal spheroids and pigmented glia’ (ALSP), which combines two distinct pathologic entities: hereditary diffuse leukoencephalopathy with spheroids (HDLS) and pigmentary orthochromatic leukodystrophy (POLD) [17, 53].

CSF1R-RD typically manifests in individuals in their 4th decade and has a mean duration of 6–8 years, although the age range and progression can vary significantly [37, 58]. To date, more than 150 genetic mutations have been linked to CSF1R-RD, with the majority located in the intracellular tyrosine kinase domain of the transmembrane CSF1R protein [28, 37, 77, 80]. In this study, we present the case of a 73-year-old man who was followed for a progressive dementing disorder of unclear etiology. Two of the patient’s siblings also succumbed to a similar disease. Standard genetic testing for a leukodystrophy panel, including *CSF1R*, yielded negative results. Postmortem examination revealed the classic findings of ALSP [17, 55], including confluent demyelination of the cerebral white matter sparing the short association fibers between adjacent gyri (U fibers), axonal damage with axonal spheroids, and glial cells with cytoplasmic pigment that stains with Sudan black (lipids) and Luxol Fast Blue and Klüver–Barrera (myelin), including microglia/macrophages. Neuropathology also demonstrated intermediate Alzheimer’s disease (AD) neuropathological change. Through long-read sequencing, we identified a novel deletion in *CSF1R* involving the tyrosine kinase domain.

CSF1R is essential for microglial proliferation, phagocytosis, motility, and survival. It is activated by homodimerization and subsequent autophosphorylation by two ligands: colony stimulating factor 1 (CSF1/M-CSF), which circulates and, in the brain, is secreted by astrocytes and oligodendrocytes, and interleukin-34 (IL-34), primarily secreted by neurons in the brain [51]. Rare homozygous *CSF1R* mutations have been reported in children with leukoencephalopathy and almost complete absence of microglia [54], a phenotype also observed in *Csf1r−/−* mice [15]. The *Csf1r+/−* mouse is a model of CSF1R-RD that recapitulates demyelination and microglia-mediated inflammation [6, 7]. Studies in CSF1R-RD postmortem brain tissue have looked specifically into the molecular phenotype of microglia. They have reported an overall loss of homeostatic markers (CX3CR1 and CSF1R, P2RY12) in microglia in gray and white matter, from relatively early disease stages, and clustered distribution of activated microglia (positive for CD68 and CD163) in the white matter [1, 32]. However, questions remain regarding the variety of pathological microglia cell states in CSF1R-RD and the intercellular crosstalk through which CSF1R mutations lead to white matter degeneration.

To gain insight into the mechanisms underlying white matter degeneration in CSF1R-RD, we conducted single nuclear RNA sequencing (snRNAseq). Because our histological data showed reduced CSF1R transcripts and protein across brain regions, including areas with histologically intact white matter, we profiled multiple brain regions exhibiting varying degrees of white matter pathology. Additionally, we included frontal cortex samples from age-matched healthy controls and individuals with AD, whose AD pathology severity was similar to that of the CSF1R-RD donor, into the dataset. We identified two CSF1R-RD-associated microglia cell states in white matter: lipid-laden and inflammatory states, alongside a smaller population of peripheral monocyte-derived macrophages. Notably, we identified a failure in OPC differentiation into mature myelin-forming oligodendrocytes, potentially contributing to the phenotype of oligodendrocyte and myelin depletion in CSF1R-RD. The remaining oligodendrocytes exhibited cell stress signatures and dysregulated apoptosis-related genes. Overall, this study contributes to unraveling the complex cellular mechanisms driving white matter degeneration and highlights the critical role of microglia-oligodendroglia interactions in CSF1R-RD.

## Materials and methods

### Human postmortem tissue

Brain tissue from the CSF1R-RD donor, two AD donors, and five healthy controls, was obtained from the Stanford Department of Pathology and the Stanford Alzheimer’s Disease Research Center, UCLA Department of Pathology, and the NIH Neurobiobank (Sepulveda repository in Los Angeles and Mt. Sinai Brain Bank in New York City). AD neuropathology grading followed the current guidelines from the National Institute on Aging and Alzheimer’s Association Research Framework [26]. The CSF1R-RD and one AD donor had a Braak neurofibrillary tangle stage of IV, and sparse neuritic plaques in neocortex (Cerad C1). Relevant information such as age, sex, ethnicity, brain weight, postmortem interval (PMI), and *APOE* genotype, were recorded when available. The mean age of the donors was 73.25 years (CSF1R-RD: 73 y/o; AD donors: 81 and 83 years; healthy controls: 53–82 years). All donors except three of the controls were males. The RNA integrity number (RIN) of the tissue selected for snRNAseq ranged from 5 to 6.7 (quantified using Agilent Bioanalyzer 2100 RNA Nano chips, Agilent Technologies cat # 5067-1511).

### Long-read sequencing

Genomic DNA extracted from the cerebellum was prepared for sequencing using the V10 ligation sequencing kit from Oxford Nanopore Technologies. The prepared library was loaded on an R9.4 PromethION flow cell and sequenced to 60 Gb. Basecalling was performed in real-time with Guppy using the high-accuracy model for R9. After filtering out low quality reads, we achieved a read length N50 of 11.6 Kb and an aligned average coverage of 16.6×. High quality reads (Qscore > 7), were aligned to the GRCh38 reference with minimap2, and structural variants (SV) were called with Sniffles2. SV calls were intersected with *CSF1R* genomic coordinates to identify the 319 bp deletion.

### Immunohistochemistry

Formalin-fixed paraffin embedded brain tissue from frontal, temporal, cingulate, parietal, and occipital cortices, and cerebellum from the CSF1R-RD donor as well as multiple brain regions from healthy and AD control donors were cut at 5 µm and mounted on Superfrost Plus (Fisher Scientific, #12-550-15) slides. Sections were dried overnight at 37 °C prior to baking at 60 °C for one hour. Immediately following baking, slides were deparaffinized with xylene and dehydrated in an ethanol series. Antigen retrieval was performed on sections using ImmunoRetriever with EDTA (BioSB, 0030) in a Hamilton Beach steamer at 99 °C for 15 min. Sections were washed in 0.2% PBT (Triton) and endogenous hydrogen peroxide activity was quenched with 1% H_2_O_2_ for 30 min in the dark at room temperature. Following three 0.2% PBT washes, sections were blocked using 10% Normal Goat or Donkey serum, 2% BSA, in 0.2% PBT for one hour at room temperature. After blocking, sections were incubated with primary antibody, either Iba1 (Wako, 019-19741) or polyclonal goat anti-GPNMB (R&D Systems, AF2330) diluted 1:2,500 in 3% normal serum, 0.5% BSA, in 0.2% PBT, overnight at 4 °C. After primary antibody incubation, sections were washed three times in 0.2% PBT for ten minutes each. Following washes, sections were incubated with biotinylated secondary antibody (1:200 in 3% normal serum, 0.5% BSA, in 0.2% PBT) for 1 h at room temperature. Prior to chromogen development, sections were incubated with the avidin–biotin complex (Vector Laboratories, PK-6100) for one hour at room temperature. Diaminobenzidine (DAB) was used to visualize the staining (Sigma Aldrich, 1.02924.0001). DAB development was stopped with three washes of 0.05 M Tris pH 7.5. Following an ethanol series and xylene washes, slides were mounted using Permount Mounting Medium (Fisher Chemical, SP15-500).

### BaseScope in situ hybridization

BaseScope was employed to quantify *CSFR1* transcripts from normal and deleted alleles in postmortem tissue obtained from the CSF1R-RD donor and three healthy controls. Human BaseScope probes were acquired from ACD Bio to detect the following nucleotide sequences within the *CSF1R* gene: 1092671-C1 (Probe-BA-Hs-CSF1R-3EJ-C1) to target nucleotides 314–895 of NM_005211.3; 1162871-C2 (custom designed probe to target nucleotides 2202–2239 of NM_005211.4 with 1zz); and 1162881-C2 (custom designed to target novel junction nucleotides 2171–2265 of NM_005211.4 by partial removal of the deletion with 1zz).

Fresh frozen human brain tissue including white matter from prefrontal cortex, medial temporal lobe, occipital lobe, pons, and cerebellum, was sectioned at 14 µm with a cryostat. Slides were stored at − 80 °C until use. Sections were fixed in 4% paraformaldehyde for 15 min at 4 °C and then washed twice in 1× PBS for 5 min. Following fixation, slides were dehydrated in 50% EtOH, 70% EtOH, and 100% EtOH. BaseScope ISH was performed using the BaseScope Duplex Detection Reagent Kit (Cat. No. 323800) according to the manufacturer’s instructions. After staining, the slides were counterstained in 50% Hematoxylin staining solution (MHS16, Sigma-Aldrich) and mounted with VectaMount permanent mounting medium (Vector Laboratories).

To quantify RNA signal, we used digital images taken at 400× magnification with a Zeiss Axio Imager M2 microscope equipped with a color digital camera (Axiocam). We counted a minimum of 200 cells showing at least one RNA copy from 15 Regions of Interest (ROIs) randomly selected across three sections per brain region and case. We quantified the number of RNA copies (represented by red and/or blue dots) per cell. Subsequently, we calculated the proportion of cells containing varying numbers of dots (1–3, 4–9, 10–15, or > 15 RNA copies per cell). All parameters were consistently maintained between images to ensure unbiased detection.

### Western blot

Postmortem tissue from the white matter dissected from the prefrontal, medial temporal and occipital lobes, pons, and cerebellum of the CSF1R-RD donor and three healthy controls was homogenized and lysed in RIPA buffer (Cell Signaling Technology, Cat#9806S) in the presence of cOmplete Protease Inhibitor Cocktail (MilliporeSigma Cat#11697498001). The samples were then centrifuged at 13,000*g*, and the supernatant was stored as the soluble fraction. Total protein concentration was quantified using a Bradford protein assay in duplicate. Subsequently, the samples were separated on 4–20% Mini-PROTEAN TGX Gels (Bio-Rad Cat#4561094) and then transferred to PVDF membranes (Bio-Rad Cat#10026934). The membranes were blocked with 5% skimmed milk in Tris Buffered Saline + 0.1% Tween 20 (TBST) and incubated with anti CSF-1R/M-CSF-R antibody (1:1000, CST #3152) and anti-beta Actin (1:25,000, Abcam Cat#ab49900) at 4 °C overnight. Then, they were washed and incubated with horseradish peroxidase-conjugated (HRP) conjugated secondary antibody for 1 h at RT. Finally, the membranes were visualized with ECL substrate (ThermoFisher Cat#32209).

### Single-nucleus isolation from postmortem fresh frozen brain tissue

We isolated nuclei from fresh-frozen brain tissue blocks obtained from the CSF1R-RD donor (white and gray matter from prefrontal cortex, and white matter from medial temporal lobe, parietal cortex, and occipital cortex), and controls (white and gray matter from prefrontal cortex). The fresh-frozen brain tissue blocks stored at − 80 °C were equilibrated in a cryostat at − 19 °C for at least 30 min prior to use. The gray or white matter was dissected under a stereomicroscope, and then chopped it into small pieces (< 1 mm^3^) with a chilled razor blade. To inhibit RNA degradation, all procedures were conducted on ice under RNase-free conditions. For tissue homogenization, a Kimble Kontes all-glass tissue grinder was utilized (Kimble, 885300-0007). Each tissue sample was dissociated using 2.4 mL of homogenization buffer containing 10 mM Tris pH 8, 5 mM MgCl_2_, 25 mM KCl, 250 mM sucrose, 1 μM DTT, 0.5× protease inhibitor (cOmplete, Roche #4693159001), 0.2 U/μL RNase inhibitor, and 0.1% Triton X-100. The homogenates were subsequently filtered through a 40-μm cell strainer, transferred into 1.5-mL Eppendorf tubes, and centrifuged at 1000×*g* for 8 min at 4 °C. The supernatant was aspirated and discarded, and the pellets were resuspended in 450 μL of cold homogenization buffer. Further clean-up was carried out using iodixanol gradient centrifugation. An equal volume (450 μL) of 50% v/v iodixanol solution (41.25 mM sucrose, 24.75 mM KCl, 4.95 mM MgCl_2_, 9.9 mM Tris pH 8, 50% w/v iodixanol) was added to the homogenate and gently mixed with a pipette. The mixture was then transferred to a 2-mL Eppendorf tube containing 900 μL of cold 29% iodixanol solution (129.17 mM sucrose, 77.5 mM KCl, 15.5 mM MgCl_2_, 31 mM Tris pH 8, and 29% w/v iodixanol) by slow layering on the top. The tubes were centrifugated at 13,500×*g* for 20 min at 4 °C, resulting in the sedimentation of nuclei at the bottom. The supernatant and top layer of myelin and cell debris were removed and discarded. The nuclei pellets were then detached with a small amount (~ 50 μL) of immunostaining buffer (0.1 M PBS; pH 7.4, 0.5% bovine serum albumin [BSA], 5 mM MgCl2, 2 U/mL DNAse I, 0.2 U/μL RNase inhibitor), transferred to clean tubes, and gently resuspended in a total volume of 200 μL of immunostaining buffer. After a 15-min incubation with immunostaining buffer at 4 °C, with gentle rocking, primary antibodies (NeuN; 1:1000, Millipore cat#MAB377; and/or PAX6; 1:500, Biolegend, 901301) were added, and the suspension was further incubated for 40 min at 4 °C with gentle rocking. Subsequently, an equal volume (500 μL) of immunostaining buffer was added, and the tubes were inverted several times before being centrifuged at 500×*g* for 5 min at 4 °C. The supernatant was removed, and the pellets resuspended in 600 μL of immunostaining buffer. Secondary antibodies (Alexa Fluor 488, Alexa Fluor 647) and a nuclear stain (Hoechst 34580; 1:1000 of 2.5 mg/ml stock, Invitrogen cat# H21486) were subsequently added, and the solutions incubated for 30 min at 4 °C with gentle rocking. Microscopic evaluation of the number and morphology of the nuclei was conducted after each critical step and immediately before FANS.

### Glia enrichment by fluorescence-activated nuclei sorting (FANS)

FANS was employed to enrich single nuclei from glia. We used a Sony SH800 cell sorter with a 100-μm microfluidics sorting chip. The primary laser, at 488 nm, was utilized for the generation of forward scatter (FSC) and back scatter (BSC). Secondary lasers emitting at 405, 488, and 638 nm were utilized to detect Hoechst 34580, Alexa Fluor 488, and Alexa Fluor 647, respectively. The FSC versus BSC gates were set with permissive limits, discarding the smallest debris and largest particles. Hoechst 34580 fluorescence was used to discriminate single nuclei from doublets and clumps. We sorted Hoechst 34580 positive singlets from CSF1R-RD white matter. For CSF1R-RD gray matter, we sorted non-neuronal (NeuN-negative) nuclei. For healthy and AD control tissue, we sorted the PAX6-positive, NeuN-negative population singlets. Nuclei were collected in collection buffer (0.1 M PBS pH 7.4, 0.1 U/μL RNase inhibitor, 1% BSA) into BSA-coated Eppendorf tubes.

### snRNAseq of postmortem human brain nuclei

snRNAseq of postmortem human brain nuclei was conducted using the 10× Genomics Chromium Single Cell 3’ v3 assay. The input single nuclear suspensions were centrifuged at 400×*g* for 5 min at 4 °C to achieve a concentration of ~ 350 nuclei per μL, determined using a hemocytometer. On average, ~ 12,500 nuclei were loaded to capture around 5,000 nuclei per sample, with an expected capture efficiency of ~ 40%. cDNA amplification and library construction were performed following the manufacturer’s instructions.

The paired-end libraries were sequenced on Novaseq 6000. A total of 12 samples were sequenced in 3 batches. For each sequencing batch, the concentration of each sample was normalized to the total number of nuclei to ensure similar numbers of reads per nucleus. Nuclei were sequenced to a depth of ~ 75,000 reads per nucleus.

### Preprocessing, quality control, and integration of snRNAseq data

The paired-end raw sequence reads were preprocessed using the Kallisto bustools package (kbpython:0.26.0). An alignment index was constructed based on the human reference pre-mRNA (GRCh38, Ensembl 105). Using the Lamanno workflow, we generated separate count matrices for spliced and unspliced transcripts. These matrices were then merged to obtain the total nucleus count matrix. Downstream analysis, including quality control, integration, cell type annotation, and differential gene expression, was performed using the nuclear transcript counts.

Empty droplets were removed by comparison with ambient RNA levels using the DropletUtils [21, 42] package. Droplets with FDR > 0.05 were removed, and 80,864 non-empty droplets were retained. To identify potential doublets, we used the DoubletFinder [46] package version 4.2. An average doublet rate of 2.53% per sample was detected. The identified doublets were labeled and retained during batch correction and data integration. Following clustering and major cell type annotation, we identified two clusters of doublets based on high doublet scores and the presence of markers specific to more than one major cell type. These clusters, containing 721 nuclei, were excluded from downstream analysis. Four additional clusters driven by low gene count nuclei, containing 15,668 nuclei, were also excluded from the analysis.

We used Scanpy in the python package [78] version 3.9.1 to analyze raw count data. First, nuclei expressing less than 100 genes were removed from the analysis. Counts were normalized and log-transformed. Highly variable genes were identified using default parameters and a dispersion threshold of 0.5. Principal Component Analysis (PCA) was applied to reduce dimensionality, and the first 50 principal components were computed. Samples were integrated using Harmony [38] with the Donor used as batch effect. After integration, the neighborhood graph was computed employing 20 PCs and 15 neighbors. Clustering was carried out employing the Leiden algorithm with a resolution parameter of 1.0. Marker genes for each cluster were determined using the Wilcoxon rank sum test with a significance threshold of adjusted *p* value (padj) < 0.05. We then applied a more stringent cutoff of 400 for the minimum number of unique molecular identifiers (UMIs) per nuclei. The final integrated dataset included 58,447 nuclei (8724 neuronal and 49,723 non-neuronal) for downstream analysis.

### Major cell type, neuronal subtype, and glial cell state annotations

The major neuronal and non-neuronal populations were identified based on the expression of known marker genes: *SLC17A7* (excitatory neuron), *GAD1* (inhibitory neuron), *FGFR3*, *AQP4*, and *GFAP* (astrocyte), *CSF1R*, *CX3CR1*, and *CD163* (microglia), *PLP1* and MOG (oligodendrocyte), *PDGFRA* and *CSPG4* (OPC), *CLDN5* and *FLT1* (endothelial), *NOTCH3* (pericyte), and *CYP1B1* and *COL15A1* (VLMC).

Glia clusters included 18,890 astrocytes, 1736 microglia, 24,242 oligodendrocytes, and 3324 OPCs. To define cell states, each glial major cell type clusters was subset, re-integrated by donor, and re-clustered. We employed 8, 11, 8, and 8 PCs and resolution parameters of 0.3, 0.3, 0.3, and 0.2 for microglia, astrocytes, oligodendrocytes, and OPCs, respectively. Marker genes for each cluster were obtained using the Wilcoxon rank sum test with the following criteria: expressed in at least 20% of the cells in the tested cluster, log2 fold change > 0.2, and adjusted *p* value < 0.05.

### Differential gene expression and enrichment analysis

We performed differential gene expression (DGE) analysis to identify changes in gene expression between CSF1R-RD-associated clusters and healthy control clusters. We employed MAST [18] version 1.24.1 using hurdle models and adjusting by the cellular detection rates (number of genes detected per sample) and the percentage of mitochondrial genes observed in each nucleus. Genes expressed in less than 10 percent of the cells were filtered out.

Enrichment analysis for hurdle models was carried out using the bootVcov1 with 99 bootstrap replicates and the gseaAfterBoot functions implemented in MAST. Only gene sets containing at least 5 genes were considered for the analysis. Gene sets were derived from the biological process branch of Gene Ontology and Reactome (the gmt files containing the gene sets were downloaded from https://www.gsea-msigdb.org/gsea/msigdb).

### Trajectory analysis

We used scVelo [2], version 0.3.1, to infer cell trajectories in the microglia subset. We carried out filtering and normalization removing genes presenting less than 20 counts. The top 2000 highly variable genes were selected for downstream analysis. First and second-order moments were computed using 30 PCs and the 30 nearest neighbors. Transcription, splicing, and degradation rates were inferred using the recover_dynamics function. Velocities were computed for each gene using the likelihood-based dynamical model. Embedded velocity vectors and velocity umaps were generated using the velocity_embedding_stream function. Latent times were estimated for each cell employing default parameters and the tl.latent_time function. Heatmaps displaying the smoothed expression levels for the 100 genes displaying the best likelihood fits were generated using the scv.pl.heatmap. Nuclei were ordered based on their latent times. Pseudo-time analysis (a random-walk based distance measures on the velocity graph) was computed employing tl.velocity_pseudotime function without specifying root or end cells. Trajectories were additionally inferred using PAGA [79]. PAGA generates a graph-like map based on the topology of the data in which weighted edges represent the connectivity between two clusters.

## Results

### Clinical description

The proband was a 69-year-old right-handed man presenting with 1–2 years of progressive decline in memory and executive function. Behavioral symptoms included poor increased irritability and weight gain of approximately 20 pounds over 6 months with a tendency to consume excessive amounts of crackers. He had little insight into his cognitive and behavioral symptoms. His family history was notable for two of his four siblings having died with neurologic disorders. An older sister died at 66 with a diagnosis of Pick’s disease. A younger brother died at 47 with a diagnosis of primary progressive multiple sclerosis. Neither underwent autopsy. The proband’s initial cognitive exam was notable for fluent, digressive speech, mild memory trouble and impaired visuospatial function. He had some mild cogwheel rigidity in the left arm but an otherwise normal elemental neurologic examination. Neuropsychological testing revealed deficits mainly in executive function and visuospatial skills. Memory was impaired but helped by cuing.

**Fig. 1:**
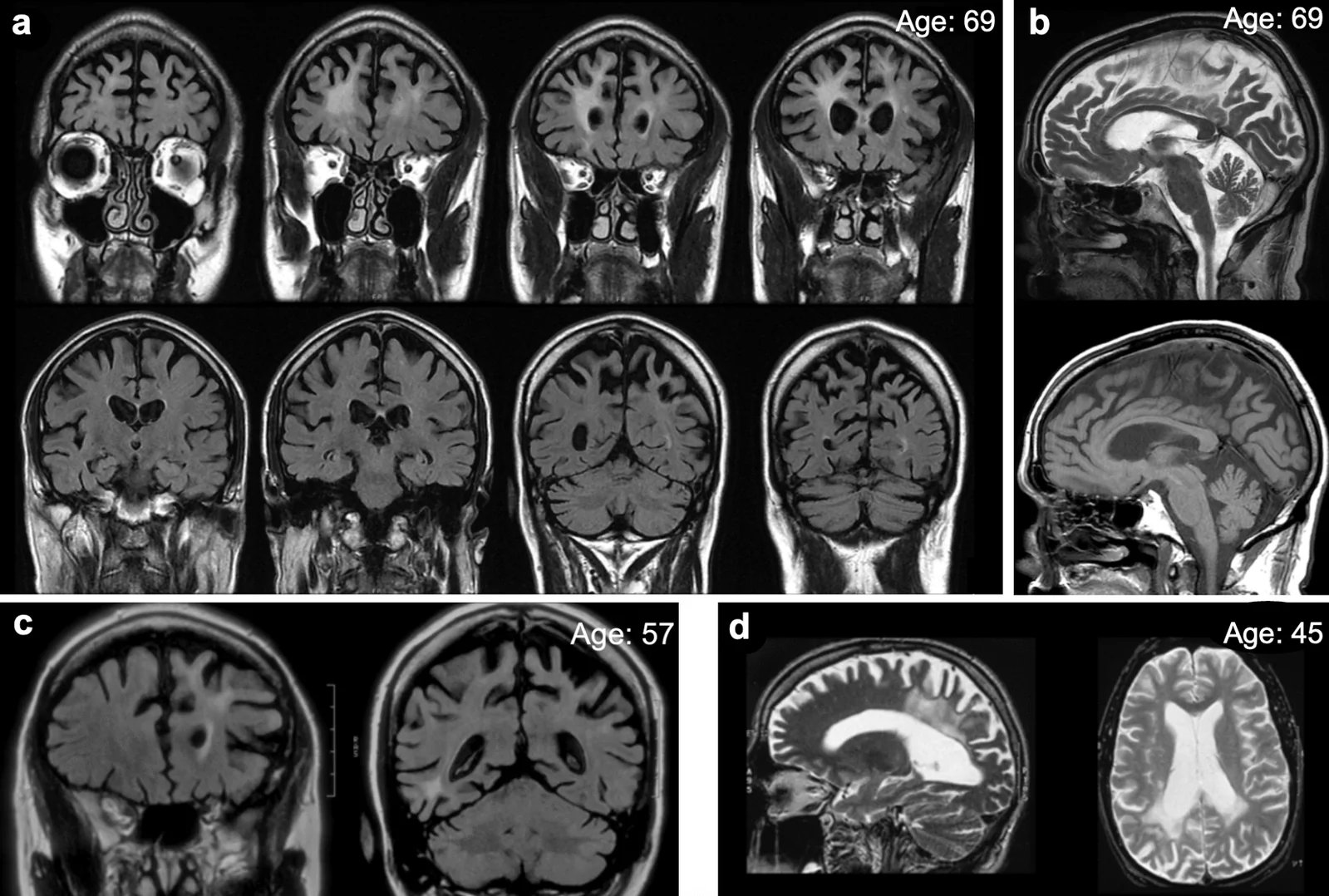
Proband’s MRI brain coronal FLAIR sequence **a** showed bilateral (right greater than left) hyperintensities in the subcortical white matter of the frontal lobes (upper row) associated with prominent bifrontal atrophy and (lower row) prominent biparietal atrophy with less impressive white matter changes. **b** Proband’s mid-sagittal T2-weighted image (top) and T1-weighted image (bottom) showed mild atrophy of the anterior aspect of the corpus callosum. **c** Affected sister’s coronal FLAIR sequence showed left greater than right frontal atrophy with mild white matter changes and prominent biparietal atrophy with focal white matter changes in the right temporal-occipital region. **d** Affected brother’s T2-weighted images showed prominent white matter hyperintensity in the posterior cingulate cortex (left side, sagittal view) and in the right greater than left lateral parietal cortex (right side, axial view). Atrophy was most prominent in the parietal lobes, with greater involvement on the right than the left

A brain MRI (Fig. 1a) demonstrated high T2 signal in the subcortical white matter of the right greater than left frontal lobes associated with bifrontal atrophy. Prominent biparietal atrophy was also noted with only minimal underlying white matter changes. Mild atrophy of the anterior component of the corpus callosum was noted. No calcifications were detected on either the MRI or CT scan at age 69. The Sundal score [72] for the proband’s MRI at age 69 was 14. The white matter findings were similar to those observed in his two siblings (Fig. 1b, c).

Routine dementia screening tests (B12, TSH, RPR) were normal or negative. An autoimmune panel was largely unremarkable other than a positive ANA test showing a speckled pattern with a 1:320 titer felt. He was seen by rheumatology who found no compelling evidence for an autoimmune disorder.

In view of the family history, genetics panels were ordered for autosomal dominant Alzheimer’s disease, frontotemporal dementia, and leukodystrophy (that included testing for *CSF1R* variants). All genetic testing was negative. The patient continued to decline gradually. He ultimately became bed-bound and died at age 73.

### Neuropathology

**Fig. 2:**
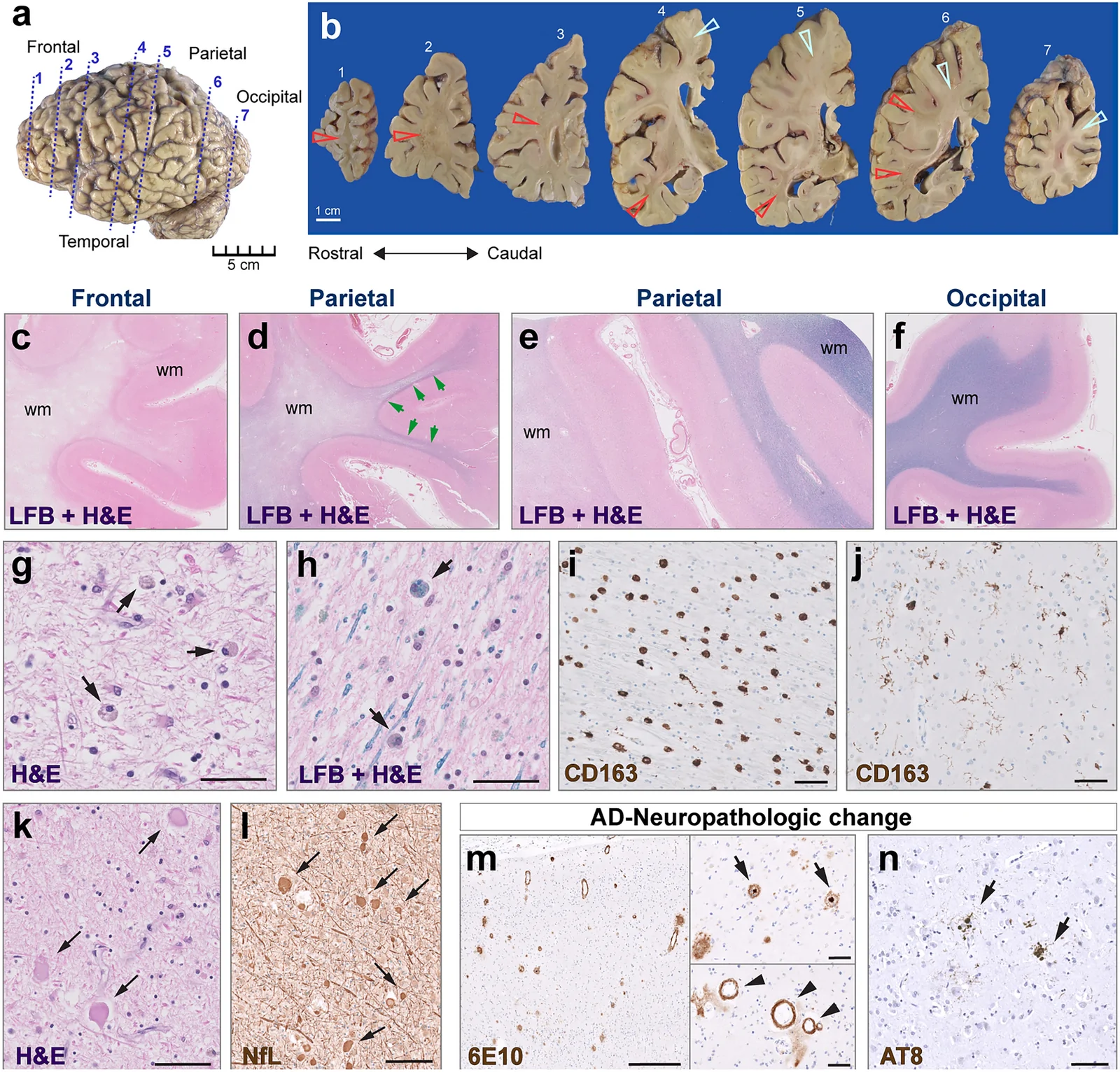
Neuropathology findings of CSF1R-RD and concurrent AD. Lateral view (**a**) of the formalin-fixed hemibrain (1355 g) shows mild to moderate diffuse cortical atrophy, greater in the frontal lobe. Coronal sections (**b**) of the left hemisphere demonstrates atrophic white matter with tan-brown discoloration involving the frontal, parietal, and temporal lobes (red arrows) and relatively preserved white matter in the pre- and post-central gyri and occipital lobes (blue arrows). Hematoxylin and eosin (H&E) and Luxol Fast Blue (LFB) stains from FFPE coronal sections through frontal (**c**), parietal (**d**, **e**), and primary visual (**f**) cortices highlight pale white matter (wm) with loss of myelin and adjacent relatively intact white matter. U fibers (green arrows) are relatively spared. White matter contains pigmented glia (myelin-laden microglia/macrophages; arrows in H&E and LFB + H&E stains) (**g**, **h**). CD163 immunostain in sections from white (**i**) and gray (**j**) matter highlights ameboid and hyper-ramified/bushy microglia, respectively. H&E and NfL immunostain demonstrate axonal spheroids (**k**, **l**; arrows). Amyloid and tau pathology (6E10 and AT8 immunostains) in parietal cortex (**m**, **n**) highlight neuritic (arrows) and diffuse plaques, p-tau threads, and cerebral amyloid angiopathy (arrowheads). Scale bars: 50 µm (**c**–**l**, n); 500 µm (**m**)

The fresh brain had a normal weight (1355 g). Grossly, it exhibited mild to moderate diffuse cerebral cortical atrophy, particularly affecting the frontal lobes, and to a lesser degree, the temporal and parietal lobes (Fig. 2a, b). The brainstem and cerebellum appeared unremarkable. Coronal sections of the cerebrum revealed severe atrophy of the white matter, especially in the frontal lobes bilaterally and symmetrically, and moderate atrophy in the temporal and the periventricular parietal regions. The white matter in these regions was soften and showed tan-brown discoloration. The U-fibers were relatively spared (Fig. 2b). The genu of the corpus callosum was atrophic, while the splenium had normal color and texture. The cortical ribbon maintained a constant and relatively preserved thickness. The deep nuclei were grossly normal.

Microscopically, confluent areas of white matter degeneration, characterized by severe loss of myelin, abundant axonal spheroids, oligodendrocyte depletion, reactive astrocytosis, and scattered pigmented microglia/macrophages, were present (Fig. 2c–l). Immunohistochemistry for CD163 highlighted reactive microglia with ameboid and hyper-ramified and bushy morphologies (Fig. 2i, j**)**. Axonal spheroids were highlighted by Neurofilaments (NfL) immunohistochemistry (Fig. 2k, l). Rare small calcifications were found in the atrophic white matter. There was minimal intraparenchymal lymphocytic infiltration or perivascular cuffing.

Additionally, Alzheimer disease neuropathologic changes (ADNC) corresponding with a NIH-NIA score of A3B2C1 were identified (Fig. 2m, n). Staining for Aβ revealed amyloid deposition in the cerebral cortex, basal ganglia, and brainstem, consistent with Thal phase 4. Tau staining revealed neurofibrillary tangles (NFTs) in the entorhinal cortex and hippocampus, and neuropil threads in the neocortex, consistent with a Braak NFT stage IV. Sparse neocortical neuritic plaques were consistent with an age-related CERAD score of C1. Aβ immunostaining also highlighted leptomeningeal and cortical arterioles and capillaries, consistent with mild cerebral amyloid angiopathy (CAA).

No significant vascular injury or hippocampal sclerosis was identified, and there was no evidence of TDP-43 inclusions or Lewy body disease. Overall, neuropathology revealed classic findings of CSF1R-RD and concurrent intermediate ADNC.

### A novel *CSF1R* partial deletion discovered by long-read sequencing

**Fig. 3:**
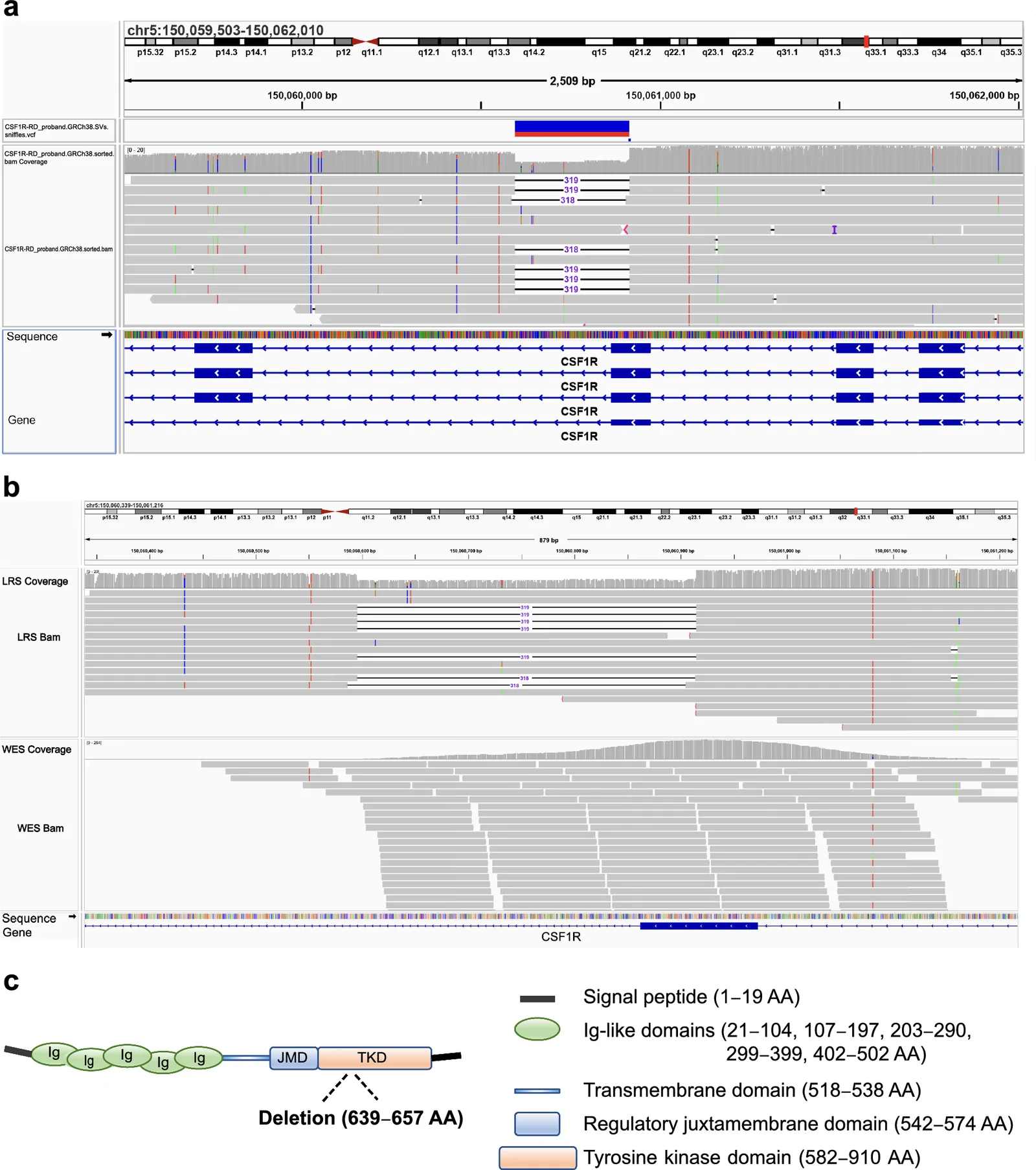
Long-read sequencing revealed a novel 319 bps deletion in *CSF1R* (**a**) that was not detected by short-read exome sequencing (**b**)**.** The CSF1R protein (972 amino acids) consists of a signal peptide, five repeated immunoglobulin-like domains, a transmembrane domain, a regulatory juxtamembrane domain, and a tyrosine kinase domain (**c**). The deletion is predicted to map within the tyrosine kinase domain (from amino acids 639 to 657)

Given the neuropathologic diagnosis of ALSP and negative results on standard genetic testing for leukodystrophy, we conducted both short-read whole-exome sequencing and long-read sequencing focusing on the *CSF1R* gene. The whole-exome sequencing did not reveal any candidate variants of interest. With long-read sequencing we identified a 319 bp deletion in *CSF1R* (GRCh38 chr5:150060596–150060915) **(**Fig. 3a**).** Even when focusing specifically on this region of the whole-exome sequencing data, the deletion was not detectable (Fig. 3b). Upon aligning the positional information of the deletion, we determined that the deletion is located on exon 14, corresponding with amino acids 639–657, which are located within the tyrosine kinase domain **(**Fig. 3c**).**

### Decreased expression of CSF1R transcripts and protein across brain regions in CSF1R-RD

**Fig. 4:**
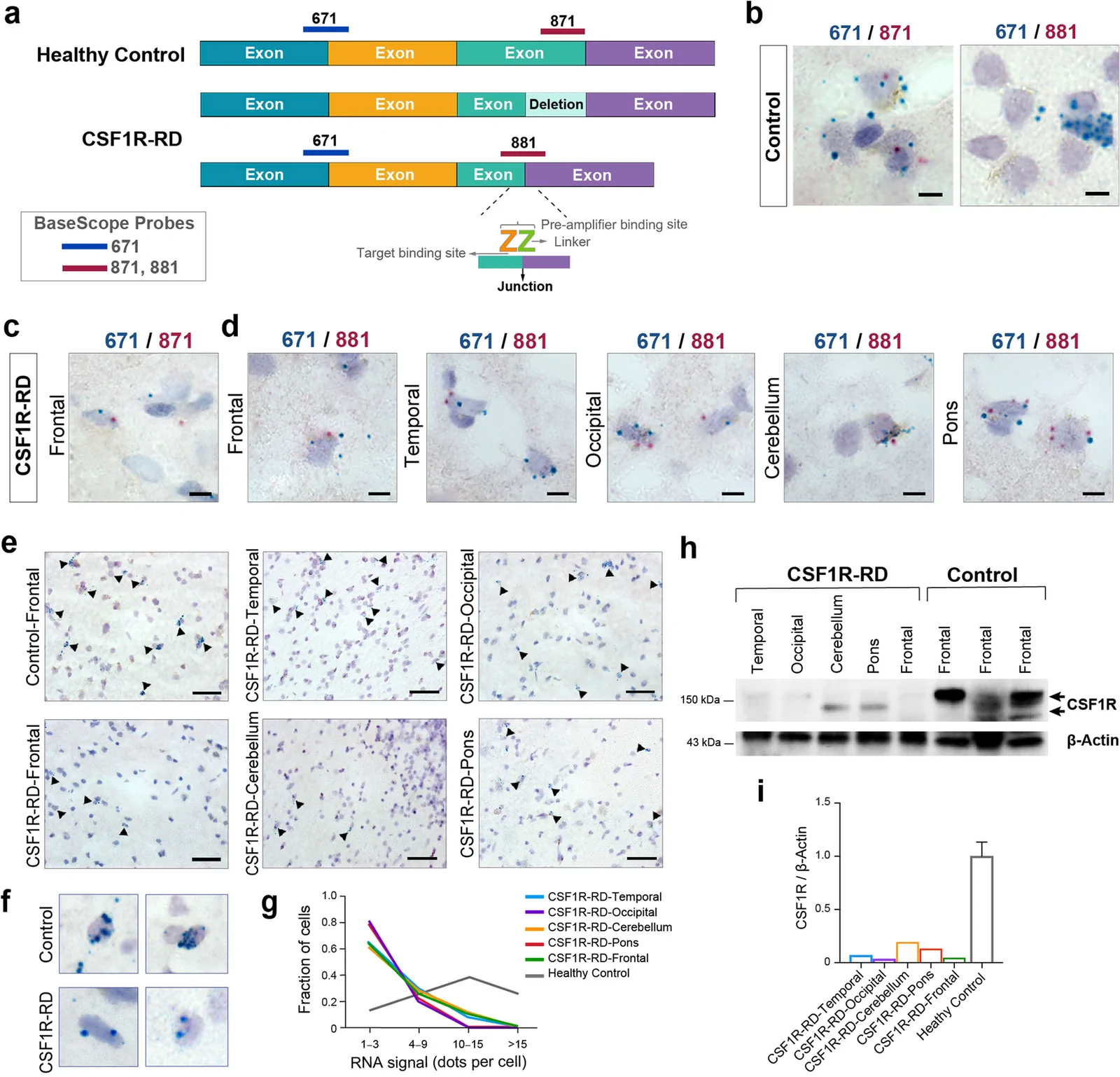
Decreased *CSF1R* transcripts and CSF1R protein across brain regions in CSF1R-RD. BaseScope probes (**a**) were designed to detect *CSF1R* from the deletion site (871: normal allele; 881: deletion allele) and included a control probe outside of the deletion site (671) detecting both alleles. Representative BaseScope images from a healthy control (**b**) show 671/871 double-positive cells and no stain for the deletion (881) probe, and from the CSF1R-RD donor show 671/871 and 671/881 double positive cells (normal and deletion alleles, respectively) from frontal (**c**), temporal, occipital, cerebellar and pontine (**d**) white matter. Low (**e**) and high (**f**) magnification views of white matter show cells with various *CSF1R* 671 signal levels (arrowheads) across brain regions from healthy controls and CSF1R-RD. **g** Quantification of transcript levels shows the fraction of cells containing either 1–3, 4–9, 10–15, or > 15 copies per cell. Each color represents a brain region. **h** Western blot demonstrates decreased CSF1R in white matter across brain regions in CSF1R-RD, compared with frontal white matter from three healthy controls. The two bands in controls represent immature and mature forms of CSR1R. CSF1R expression was nearly absent in frontal, temporal, and occipital white matter, and greatly reduced in the cerebellum and pons (**i**). Scale bars: 10 µm (**b**–**d**); 50 µm (**e**)

Previous studies have reported decreased CSF1R RNA and protein in CSF1R-RD [32, 36]. To assess the impact of the newly identified CSF1R deletion on RNA and protein levels, we quantified *CSF1R* transcripts and protein across brain regions in CSF1R-RD and healthy control tissue. To quantify *CSF1R* transcript levels, we employed BaseScope in situ hybridization (ISH). We designed three probes to detect: the region of the deletion with (probe 881) or without (probe 871) the deletion, and a control probe located outside of the deletion site that is common to both alleles (probe 671) (Fig. 4a). We validated the absence of the deletion allele in brain tissue from healthy controls **(**Fig. 4b**)**, and confirmed heterozygosity for the deletion in tissue samples from different brain regions of the CSF1R-RD donor (Fig. 4c, d).

To determine if *CSF1R* transcript levels varied across regions with various degrees of white matter degeneration, we compared the expression of *CSF1R* (probe 671) in the white matter from the frontal (most affected), medial temporal, and occipital lobes (intermediate), as well as the cerebellum and pons (relatively preserved), and the frontal cortex from three healthy controls (Fig. 4e, f). We observed a decrease in the number of RNA copies in all regions of CSF1R-RD compared to healthy controls. The decrease was comparable across regions (Fig. 4g), suggesting that decreased *CSF1R* levels precede white matter degeneration.

To quantify CSF1R protein, we performed western blot analysis of protein extracts from the same white matter regions and donors used for BaseScope ISH (Fig. 4h). We observed very low CSF1R protein levels in the CSF1R-RD cerebellum and pons, minimal levels in the temporal and occipital regions, and virtually undetectable protein in the frontal region (Fig. 4i). Overall, our data showed low levels of CSF1R RNA and protein in both affected and relatively preserved white matter, suggesting that the partial deletion of one CSF1R allele is sufficient to result in a greater than 50% decrease in CSF1R protein, even before white matter appears grossly affected.

### snRNAseq of CSF1R-RD across brain regions and integration with AD and healthy controls

**Fig. 5:**
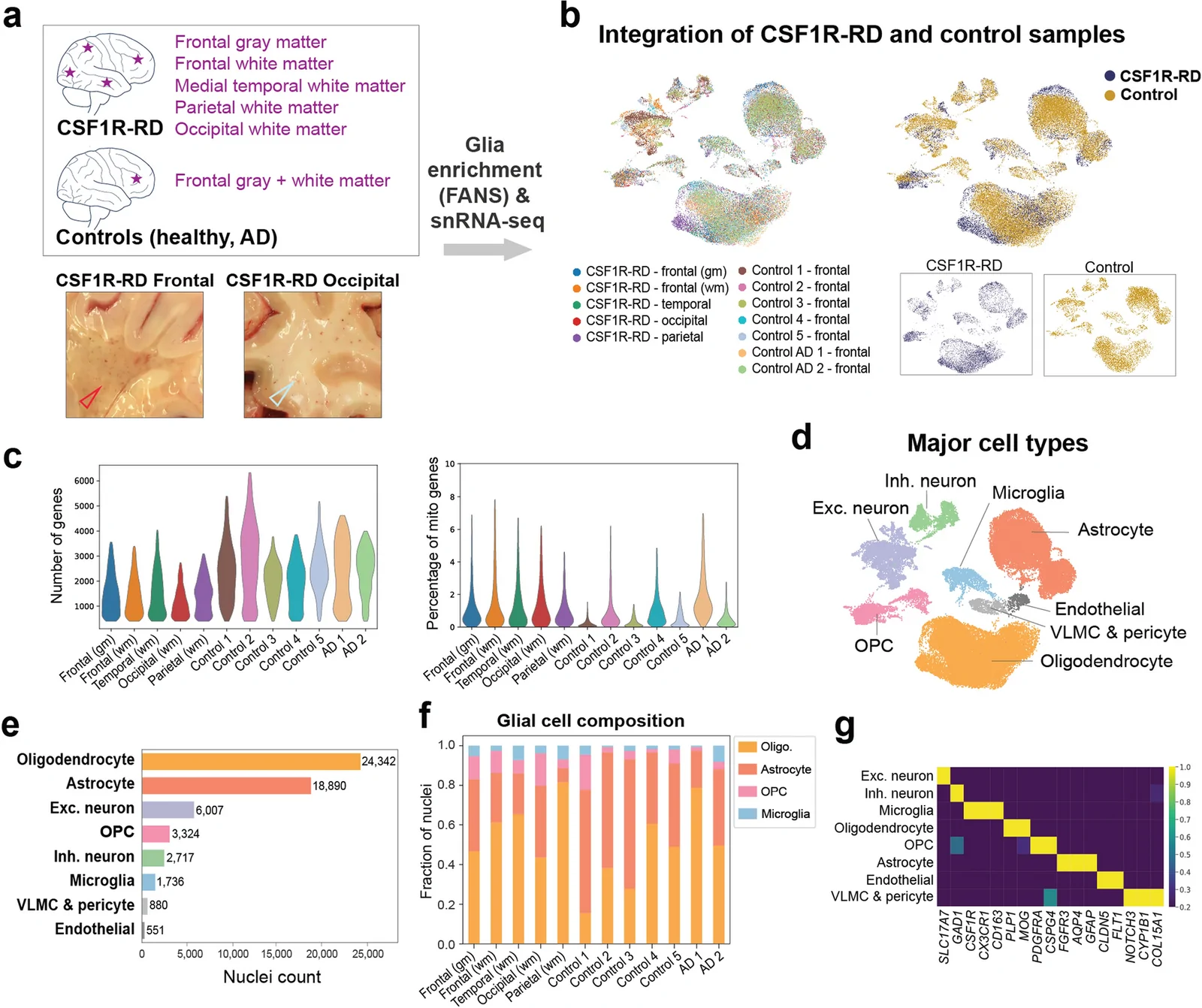
snRNAseq of CSF1R-RD and integration with control datasets. **a** Five brain regions from the CSF1R-RD donor were profiled and compared with frontal cortex from age-matched healthy control and AD donors. Representative photographs depict CSF1R-RD regions profiled that had grossly intact (occipital cortex, blue arrow) and degenerated (frontal cortex, red arrow) white matter. **b** Glial nuclei were enriched using FANS and profiled using 10x. The integrated dataset contained 12 samples (5 CSF1R-RD, 5 healthy control, 2 AD). The UMAP plots represent the contributions from CSF1R-RD and controls, as well as from each sample individually. **c** Violin plots showing the median number of genes and the percentage of mitochondrial genes within each sample. **d** UMAP showing the annotation for major cell types (excitatory and inhibitory neurons, microglia, astrocytes, oligodendrocytes, OPCs, vascular cells). **e** Bar plot showing the contribution of nuclei from each major cell to the dataset. **f** Bar plot showing the fraction of nuclei corresponding to each glial cell type within each sample. **g** Heatmap showing normalized gene expression of major cell type marker genes

snRNAseq of human postmortem tissue from demyelinating, neuroinflammatory, and neurodegenerative disorders has revealed disease-specific cell states [19, 33, 45, 48, 67, 70]. To gain insight into the glial cell types and states associated with white matter degeneration in CSF1R-RD, we profiled the frontal cortex (gray and white matter) and white matter regions of the medial temporal, parietal, and occipital cortex. Since the CSF1R-RD donor had ADNC, we included frontal cortex samples (gray and white matter) from two donors with similar ADNC (Braak NFT stage IV; Cerad C1), as well as frontal cortex samples (gray and white matter) from five age-matched healthy donors (Fig. 5a). To enrich for glia, we utilized fluorescence-activated nuclear sorting (FANS) with either negative selection for neurons (NeuN-negative) or positive selection for glia (PAX6-positive, NeuN-negative). After quality control, we obtained 58,447 high-quality nuclei (17,063 ALPS, 10,348 AD, 31,036 healthy). Dataset integration using Harmony and unsupervised Leiden clustering successfully integrated the nuclei derived from different brain regions, disease groups, and donors (Fig. 5b, c). We annotated eight major cell types: excitatory neurons (10.3%), inhibitory neurons (4.7%), oligodendrocytes (41.5%), OPCs (5.7%), astrocytes (32.4%), microglia (3%), endothelial cells (0.9%), and pericytes and VLMC (1.5%) (Fig. 5d–g). Subsequently, we subset and analyzed separately the microglia, astrocyte, oligodendroglia, and OPC populations to further characterize CSF1R-RD-associated glial cell states.

### Microglia cell states in CSF1R-RD

**Fig. 6:**
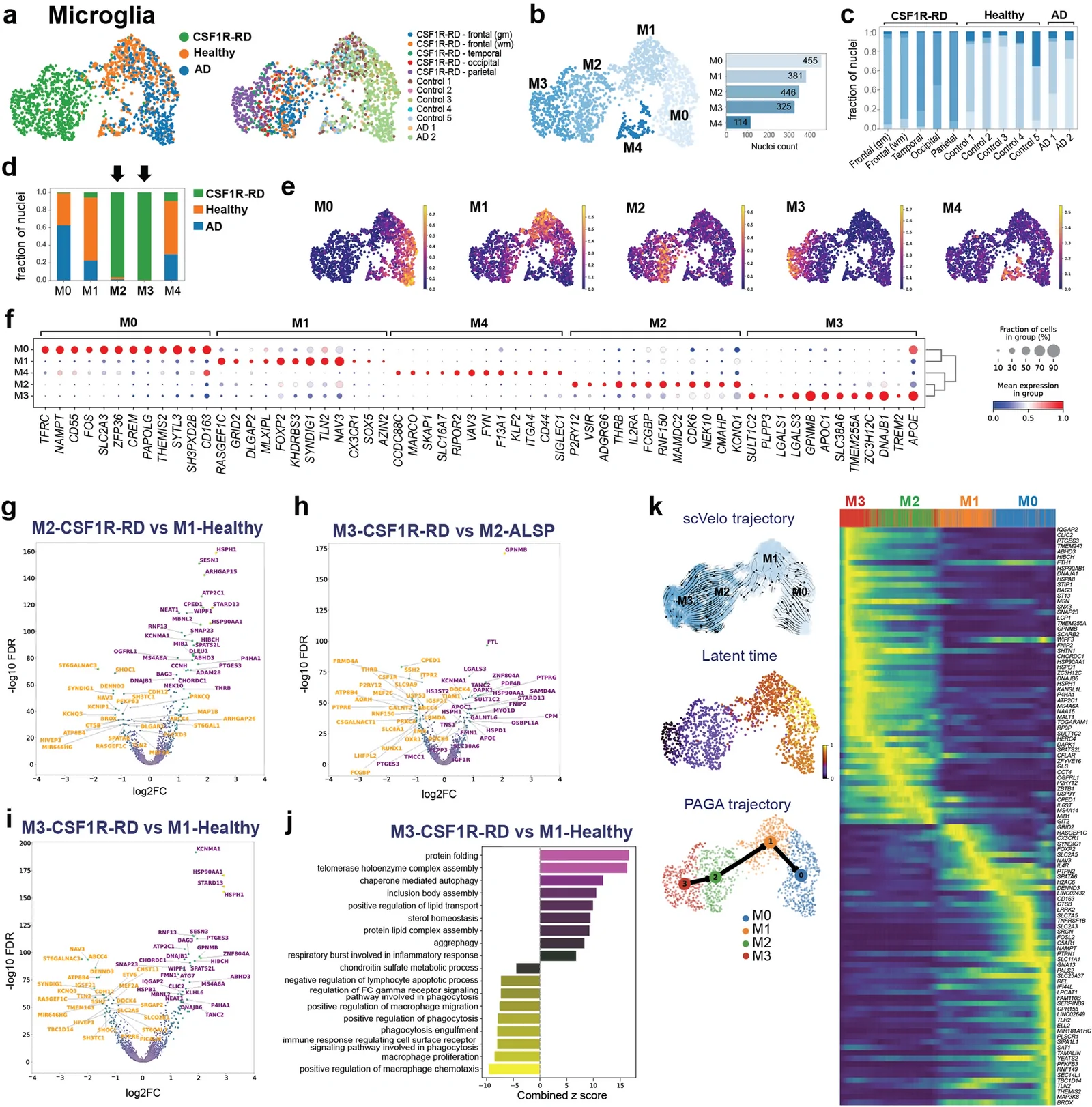
Microglial cell states in CSF1R-RD. **a** UMAP plots showing the contributions from CSF1R-RD, healthy control, and AD (left), and from each individual sample (right). **b** UMAP and bar plots showing the five annotated microglia states (M0–M4) and the numbers of nuclei profiled for each. **c** Contribution of each microglia cluster per sample. **d** Contribution of each disease group (CSF1R-RD, AD, healthy) to each microglia state cluster. M2 and M3 (arrows) represent CSF1R-RD-associated cell states. **e** Visualization of each microglia cluster-defining gene set in UMAP plots. **f** Dot plot showing top microglia state marker genes for each cluster. **g**–**i** Volcano plots depicting DE genes between the two CSF1R-RD-associated microglia states (M3 vs. M2) and between either M2 or M3 and M1-healthy controls. **j** Top biological pathways enriched in M3-CSF1R-RD compared with M1-healthy controls. The x-axis represents combined Z scores (magenta: overrepresented in M3; yellow: underrepresented in M3). **k** Microglia subset trajectory analysis. The UMAPs display the embedding streams, the pseudo-time values for each nucleus, and the PAGA cluster connectivity graph. The heatmap shows genes with the highest likelihood fits to the nuclei order based on latent times

We annotated five microglia clusters (M0–M4; Fig. 6, Supplementary Fig. 1, Supplementary Table 1). Two clusters were enriched in healthy and AD control tissues (M1 and M0, respectively). M1 was defined by known makers for homeostatic microglia (*CX3CR1*, *SOX5*, *FOXP2*, *GRID2*, *KHDRBS3*, *RASGEF1C, SYNDIG1*). M0 had marker genes for phagocytosis (*ARHGAP18, CDC42*, *CD163L1*, *CLEC7A*, *SLC11A1*, *CEBPD*, *IFI44L*, *FAM110B*), neuronal surveillance, and glycolysis [45, 57, 70]. Two clusters (M2 and M3) were specifically enriched in CSF1R-RD and corresponded with pro-inflammatory (M2) and autophagy (M3) cell states. A smaller cluster (M4) contained nuclei from both CSF1R-RD and controls and represented infiltrating monocyte-derived macrophages (expressing *CD44*, *CCDC88C*, *MARCO*, *F13A1*, and *ITGA4*) with migratory and phagocytic properties (Fig. 6a–f, Supplementary Fig. 1e).

The M2 CSF1R-RD-associated cluster specifically expressed genes involved in immune regulation, including *FCGBP* (IgG Fc Binding Protein), *FCGR3A* (CD16, Fc Gamma Receptor), *IL2RA* (CD25, Interleukin 2 Receptor), *IL18, VSIR* (known as *VISTA*), *ADGRG6*, and *MAMDC2*. A pro-inflammatory function of cells in this cluster is suggested by the expression of *IL2RA, FCGBP, VSIR, SESN3,* and *ATP2C1* (Fig. 6f, Supplementary Fig. 1c). Compared with M1-healthy, the M2-CSF1R-RD nuclei upregulated *HSPH1*, *HSP90AA1*, *ARHGAP15*, and *STARD13*, whereas the glycosylation-related gene *ST6GALNAC3* was downregulated (Fig. 6g; Supplementary Table 2).

The M3 ALPS-associated cluster specifically expressed genes involved in lipid metabolism or autophagy, including genes in previously classified lipid-processing state microglia such as *CPM*, *MGLL* and *PTPRG*, as well as *SQSTM1* (Sequestosome 1 or p62), a classical selective autophagy receptor, *GPNMB* (glycoprotein Nonmetastatic Melanoma Protein B), a marker for lipid-laden macrophages, and *PPARG, HEXA, PLPP3, HS3ST2, LGALS1* and *LGALS3* (Fig. 6f, Supplementary Fig. 1d). GSEA pathway enrichment analysis comparing M3-CSF1R-RD to M1-healthy showed high enrichment scores for protein folding, chaperone mediated autophagy, lipid transport, and sterol homeostasis, whereas phagocytosis pathways were underrepresented (Fig. 6j; Supplementary Tables 3, 4).

Inferred trajectories for microglia states M0-M3 using scVelo indicated cell transitions between M3-2 (CSF1R-RD-associated clusters) and M0 (AD-associated cluster), with M1 (homeostatic microglia) in an intermediate position. This suggests that the CSF1R-RD and AD-associated microglia clusters represent different activated microglia states evolving from M1. Genes with the highest likelihood fit and a gradual increase in expression during the transition from M1 to M3-M2 included components of the response to unfolded proteins (*HSP90AB1, DNAJA1, HSPA8, BAG3, ST13, HSP90AA1, HSPD1, HSPH1*), genes linked to lipid response metabolism and transport (*HSPA8, MSN, LCP1, SCARB2, ABHD3*), and *GPNMB*. In contrast, a gradual decrease in homeostatic genes (*GRID2, RASGEF1C, CX3CR1, SYNDIG1*, *FOXP2*) was observed when transitioning from both CSF1R-RD and AD-associated clusters to the homeostatic microglia. Gradual increases in the expression of genes linked to phagocytosis (*PLSCR1, SLC11A1, TLR2*), cytokine production (*SLC7A5, TNFRSF1B*), and microglia activation (*SLC11A1, C5AR1*) were observed in the transition from M1 to M0. Pseudo-time analysis and cluster connectivity patterns identified by PAGA supported the results from the dynamical model analysis (Fig. 6k).

**Fig. 7:**
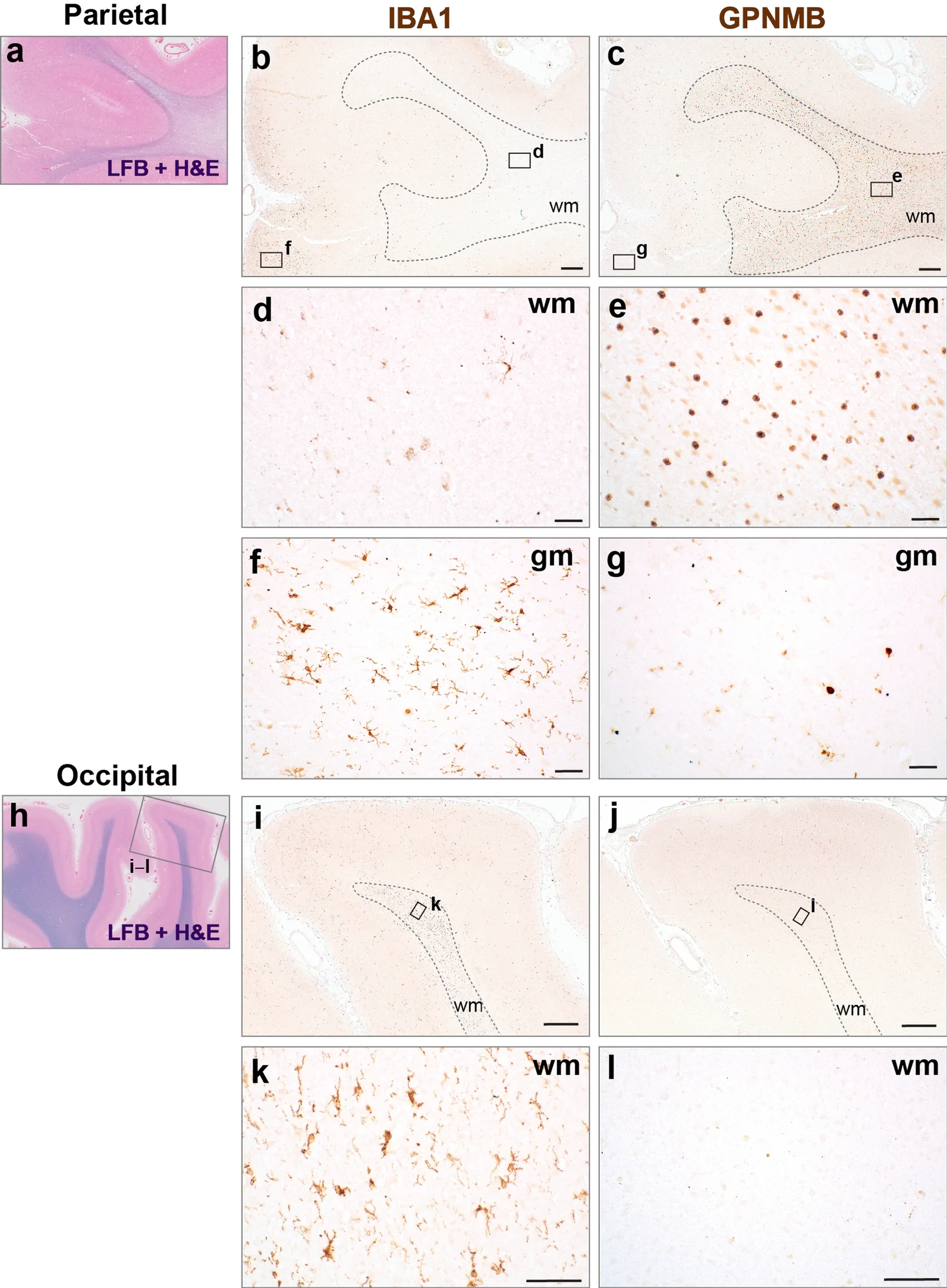
GPNMB expression in lipid-laden microglia. Immunohistochemistry for GPNMB and IBA1 in a severely affected cortical region (parietal; **a**–**g**) and a relatively preserved region (occipital; **h**–**l**). LFB + H&E stains from FFPE sections highlight white matter with severe loss of myelin (**a**) and relatively intact myelin (**h**). GPNMB + microglia are abundant in degenerated parietal white matter (**e**) and sparse or not present in parietal gray matter (**g**) and occipital white matter (**l**). Most GPNMB+ cells have round cell bodies with no processes. In contrast, IBA1+ microglia are depleted in degenerated white matter from the parietal cortex (**d**) and exhibit hyper-ramified, bushy, or rod-like morphologies in parietal gray matter (**f**) and occipital white matter (**k**). Dashed lines indicate approximate borders between gray matter (gm) and white matter (wm). Scale bars: 500 µm (**b, c, i, j**); 50 µm (**d**–**g; k, l**)

The M3 cluster exhibited the lowest levels of *CSF1R* and the highest *TREM2* gene expression (Supplementary Fig. 2), potentially indicating a compensatory response, as *TREM2* and *CSF1R* interact to promote microglial survival [11]. Among the genes that best distinguished M3 from the other microglial states was *GPNMB* (Fig. 6f, h, k), a marker for lipid-laden macrophages across tissues and diseases with autophagic and anti-inflammatory functions [76]. GPNMB has also been shown to be increased in brain tissue and CSF from AD patients, where it is expressed in a subset of microglia clustering around amyloid plaques [25, 66, 81]. Immunohistochemistry for GPNMB confirmed expression in lipid-laden microglia in CSF1R-RD (Fig. 7). GPNMB+ microglia displayed round cell bodies with few or no cell processes (Fig. 7e) and were abundant in white matter from atrophic and severely degenerated regions, including frontal, parietal, and cingulate, and sparse or absent in gray matter and in white matter with relatively intact myelin (Fig. 7, Supplementary Fig. 3).

### Astrocyte cell states in CSF1R-RD

We annotated four astrocyte clusters (A0–A3; Supplementary Fig. 4). A0 comprised the largest proportion and represented homeostatic astrocytes (high *SLC1A2* and low *GFAP*), while the other clusters represented reactive cell states previously characterized in neurodegenerative and other neurological conditions [10, 16, 41, 64]. A1 showed high expression of *IFI6, OSMR, and CHI3L1*. A2 exhibited high expression of *GRIA1*. A3 showed high levels of *CD44*, *VCAN, and AQP1*. Notably, the receptor CD44, predominantly expressed by fibrous astrocytes in white matter, has been shown to bind GPNMB, reducing NFκB activation and subsequent inflammatory responses in microglia/macrophages [52]. CSF1R-RD white matter from the parietal, temporal, and occipital cortex had the largest proportion of the *CD44*-containing astrocytes in A3 (Supplementary Fig. 4c, e).

### OPC and oligodendrocyte cell states in CSF1R-RD

**Fig. 8:**
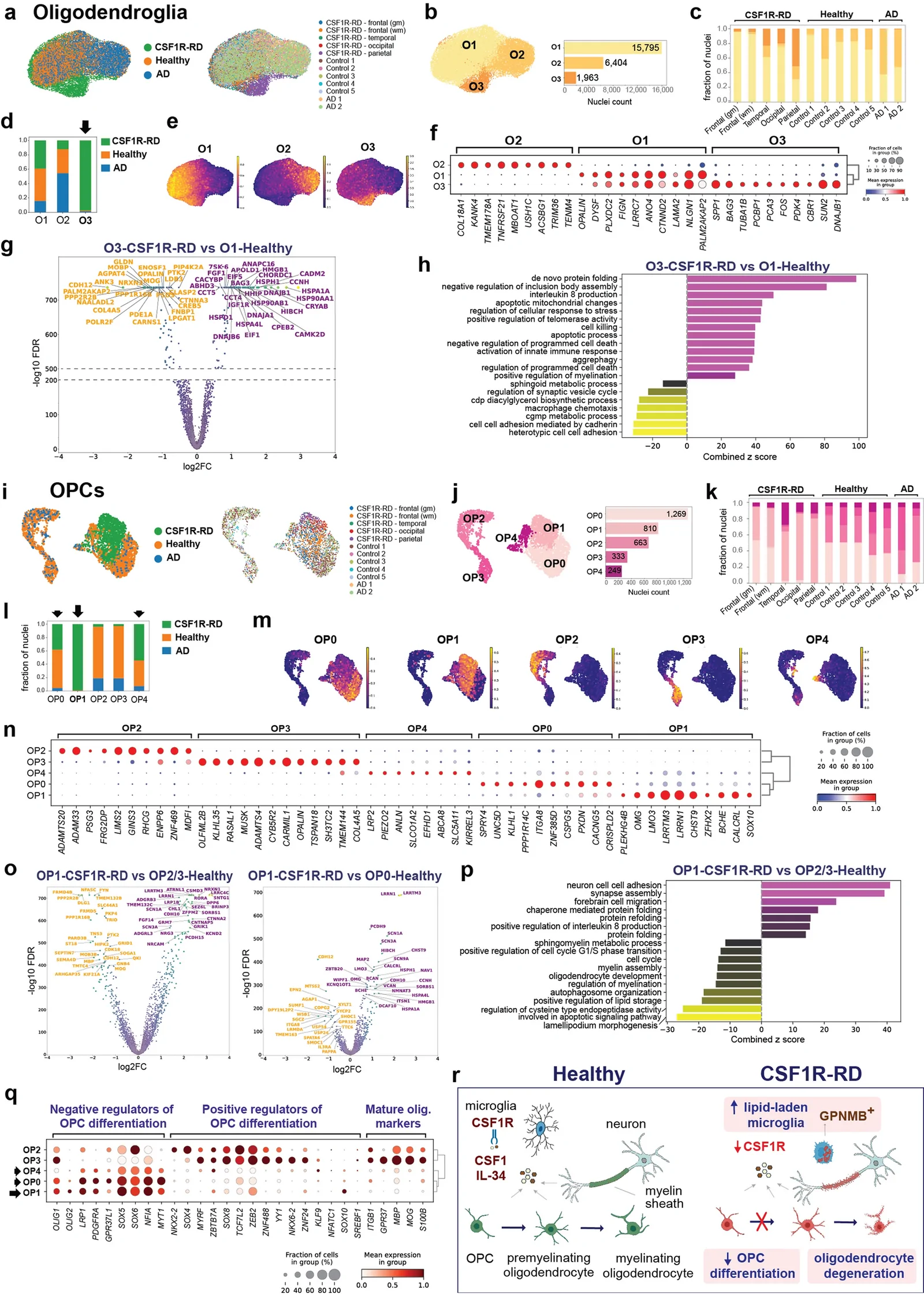
Oligodendrocyte and OPC cell states in CSF1R-RD.** a**–**h**, Oligodendrocyte cell states. **a** UMAP plots showing the contributions from CSF1R-RD, healthy control, and AD (left), and from each individual sample (right).** b** UMAP and bar plots showing the three annotated oligodendrocyte cell states (O1–O3). **c** Contribution of each oligodendrocyte state per sample. **d** Contribution of each disease group (CSF1R-RD, AD, healthy) to each oligodendrocyte state cluster. O3 (arrow) represents an CSF1R-RD-associated cell state **e** Visualization of each oligodendrocyte cluster-defining gene set in UMAP plots. **f** Dot plot showing top oligodendrocyte state marker genes for each cluster. **g** Volcano plot depicting DE genes between O3-CSF1R-RD and O1-healthy controls. **h** Top biological pathways enriched in O3-CSF1R-RD compared with O1-healthy controls. The x-axis represents combined Z scores (magenta: overrepresented in O3; yellow: underrepresented in O3). **i**–**q** OPC states. **i** UMAP plots showing the contributions from CSF1R-RD, healthy control, and AD (left), and from each individual sample (right). **j** UMAP and bar plots showing the five annotated OPC states (OP0–OP4) **k** Contribution of each OPC state per sample. **l** Contribution of each disease group to each OPC state cluster. The OP1 cluster is derived almost exclusively from CSF1R-RD; CSF1R-RD-derived nuclei also contribute to clusters OP1 and OP2 (arrows) **m** Visualization of each OPC cluster-defining gene set in UMAP plots. **n** Dot plot showing top OPC marker genes for each cluster. **o** Volcano plot depicting DE genes between the CSF1R-RD associated cluster OP1 and combined OP2 and OP3 healthy controls (left), and between OP1-CSF1R-RD and OP0-healthy controls. **p** Top biological pathways enriched in OP1-CSF1R-RD compared with combined OP2 and OP3 healthy controls. The x-axis represents combined Z scores. **q** Relative expression of genes known to be associated with the differentiation of OPCs into myelin-forming oligodendrocytes for each cluster. OP2 and OP3 represent maturing OPCs. The CSF1R-RD disease associated clusters OP1, OP0 and OP4 show increased expression of negative regulators of OP differentiation and decreased expression of positive regulators of OP differentiation.** r** Model of CSF1R-RD glial disease pathogenesis. In the healthy brain, activation of CSF1R signaling by the ligands CSF1 (secreted by oligodendrocytes, astrocytes, and OPCs) and IL34 (secreted mainly by neurons) regulates microglia homeostasis and myelin maintenance. In CSF1R-RD, CSF1R dysfunction results in pro-inflammatory and autophagy microglia states, myelin phagocytosis, and arrest in OPC differentiation. GPNMB is a marker for lipid-laden microglia (“pigmented glia”) in ALSP/CSF1R-RD

We annotated three oligodendrocyte clusters (O1–O3; Fig. 8a–h, Supplementary Fig. 5). O1 and O2 represented two widely recognized molecular oligodendrocyte cell subtypes, expressing *OPALIN* (O1) or *COL18A1* (O2) [30, 64, 67], whereas O3 represented an CSF1R-RD-associated state with high expression of stress-response, immune-response, and apoptosis-related genes (Fig. 8d–f).

The CSF1R-RD-associated cluster O3 expressed high levels of genes encoding heat shock proteins (*DNAJB1, DNAJB6, HSP90AA1, HSP90AB1, HSPA1A, CRYAB*), the iron chaperon *PCBP1*, the co-chaperone *BAG3*, the immediate early gene *FOS*, *TNFRSF12A* [56] (Tumor Necrosis Factor Receptor Superfamily Member 12A), and regulators of myelination and remyelination (*SPP1, BEX1*) (Fig. 8f, g). Pathway enrichment analysis comparing O3-CSF1R-RD to O1-healthy showed high enrichment scores for de novo protein folding, cell death and apoptosis related pathways, and interleukin 8 production (Fig. 6j).

Among the five clusters annotated for OPCs (OP0–OP4; Fig. 8i-q), OP1 showed the highest association with CSF1R-RD, while OP2 and OP3 primarily originated from healthy controls. OP0 and OP4 contained nuclei from both CSF1R-RD and controls samples (Fig. 8l). The CSF1R-RD-associated OP1 showed an upregulation of *LRRN1*, *LRRTM3*, *SCN1A*, *SCN3A*, and *CDH10* compared to OP2/OP3 or OP0 (Fig. 8o). Pathway enrichment analysis showed an overrepresentation of neuron cell–cell adhesion, synapse assembly, protein folding, and positive regulation of interleukin 8 production in OP1- CSF1R-RD compared to the healthy OP2/OP3 nuclei. Conversely, oligodendrocyte development, myelination, and cell cycle related pathways were underrepresented (Fig. 8p). Overall, CSF1R-RD-associated OPCs showed signatures suggesting a loss in their ability to differentiate into mature oligodendrocytes (Fig. 6q), as indicated by the upregulation of genes known to negatively regulate OPC differentiation (*LRP1, PDGFRA, SOX5, SOX6, NFIA*), downregulation of genes known to positively regulate OPC differentiation (*NKX2-2, NKX6.2, SOX4, SOX8, TCF7L2, YY1, ZNF488*), and decreased expression of markers of mature oligodendrocytes (MBP, MOG, S100B, ITGB1).

In summary, our findings support a model in which decreased and/or aberrant CSF1R expression in microglia leads to their differentiation into pro-inflammatory and autophagocytic cell states. This process promotes demyelination and neuroinflammation while inhibiting OPC differentiation into myelinating oligodendrocytes (Fig. 8r).

## Discussion

CSF1R-RD is clinically heterogeneous yet unified by genetic alterations in the *CSF1R* gene, which are typically inherited in an autosomal dominant manner, though sporadic mutations can be found in ~ 40% of cases [37]. The mean age of onset is around 40 years but has a wide range from 18 to 78 years, and the mean age at death is approximately 50 years (range 23–84 years). The mean disease duration is 6.8 years (range 1–29 years) [35]. Though age at onset may be earlier in women than men, disease duration and survival appear to be the same between sexes [50]. The disease penetrance increases with age for carriers of the mutation, with 10% at age 27 years, 50% at age 43 years, and 95% at age 60 years [35, 37].

Phenotypical presentation is variable and may easily be mistaken for other neurological diseases including multiple sclerosis, frontotemporal dementia or Lewy Body Disease [37, 49, 58, 68, 71]. There are two major phenotypes: cognitive predominant and motor predominant. The proband in this study had the cognitive predominant subtype. The most frequent initial presentation is early-onset cognitive decline and diverse neuropsychiatric symptoms. This is followed in frequency by motor symptoms, including parkinsonian symptoms, pyramidal signs, gait disturbances, and then bulbar signs (dysarthria, dysphagia) [27, 37, 82]. Younger women in their 20 s are more likely to present with early motor symptoms, which may be misdiagnosed as multiple sclerosis during the earliest phases of the disease [29, 35, 37, 61, 82]. Cortical symptoms have also been described, including apraxia, aphasia, and seizures in one-third of patients [35]. Stroke-like episodes, sensory symptoms, dizziness, and fatigue are also reported [35, 40, 59]. Progression is relatively rapid, with loss of independence and death occurring in a matter of a few years [35]. Though nonspecific, some imaging findings may help in the diagnosis: On non-contrast head CT, small calcifications can be found, even at asymptomatic stages, particularly in the frontal subcortical white matter, sometimes taking the characteristic stepping-stone appearance in the frontal pericallosal region as seen on sagittal views [8, 35]. Calcifications can also be found to a lesser extent in parietal subcortical white matter. MRI brain findings without contrast include bilateral confluent white matter hyperintensity, not necessarily symmetrical, sparing the U-fibers, and predominant in the frontal and parietal regions, with atrophy following the same pattern [8, 35, 59]. The lesions are nonenhancing after gadolinium injection. DWI changes correlating with ADC (diffusion-restricted lesions with reduced apparent diffusion coefficient) can be observed and may reflect intramyelinic edema [1, 50]. This can persist for several months, which helps distinguish it from acute ischemic stroke. Lateral ventricles may appear larger than expected for the patient’s age [37]. Early corpus callosum abnormalities may be seen, with thinning and signal changes described [59]. Other nonspecific findings can include abnormal signaling in the pyramidal tracts and diffusion-restricted lesions in the white matter. All these findings are supportive of the diagnosis [58, 69].

Confirmation of the diagnosis is achieved through genetic testing. However, some families with typical clinicopathological features of CSF1R-RD test negative for *CSF1R* mutations on Sanger or exome sequencing [13, 28]. This was the case for the patient in this study, where, using long-read sequencing, we discovered a novel 319 bp deletion involving exon 14, located in the tyrosine kinase domain, which is encoded by exons 12–21. This deletion is large enough to be classified as a structural variant. A recent mutational analysis of *CSF1R*, which included long-read sequencing performed on a consecutive series of 100 unrelated patients with adult-onset leukoencephalopathy, identified 15 cases with *CSF1R* mutations. Among these were two novel large partial deletions of *CSF1R*: 18,927 bp, involving exons 18–22, and 4,071 bps, involving exons 16–22, both of which involve the tyrosine kinase domain [28]. Current diagnostic tools often focus on smaller alterations, such as single nucleotide variants (SNVs) and deletions-insertions shorter than 50 bps (indels). Therefore, comprehensive mutational analysis in patients with leukoencephalopathy is crucial for accurate genetic diagnosis and for broadening our understanding of the genetic variations associated with CSF1R-RD.

Both haploinsufficiency and dominant negative models have been proposed for CSF1R deficiency in CSF1R-RD [24, 32, 35, 62]. In the dominant negative model, the protein produced by the allele with the deletion may form dysfunctional dimers, either homodimers or heterodimers with normal CSF1R, interfering with the functions of the tyrosine kinase receptor or the signaling complex [60, 62]. Our quantitative analysis of CSF1R protein from CSF1R-RD white matter tissue revealed a significant reduction exceeding 50%, which may result from a dominant negative effect. Quantification of *CSF1R* transcripts from CSF1R-RD white matter tissue using BaseScope also revealed markedly reduced transcript levels. However, *CSF1R* transcript levels measured by snRNAseq were heterogenous among microglia cell states. Although the interpretation of CSF1R expression data is limited by the low number of microglial cells from a single donor, our analyses suggest a reduction in both RNA and protein levels, consistent with previous studies [1, 32, 36, 63]. This reduction may result from protein degradation, dominant negative effects, transcriptional dysregulation, and/or RNA instability. Notably, we observed this reduction in both atrophic and grossly intact white matter. Thus, white matter degeneration may be delayed by years even with reduced levels of CSFR1 in CSF1R-RD. Our analysis represents a step toward understanding the dynamics of CSF1R transcripts and protein expression in aging patients with CSF1R-RD. However, substantial additional research is needed to identify the factors that may drive decompensation and accelerate disease progression.

CSF1R-RD represents a type of primary microgliopathy, as CSF1R is exclusively expressed in the brain by microglia/macrophages, crucially modulating microglial homeostasis [17, 35, 75]. While microglia are dispensable for myelin sheath formation in development, they play essential roles in myelin maintenance and preventing its degeneration [47]. Microglia survey the CNS environment and clear debris through phagocytosis, contributing to myelin maintenance during aging, neurodegeneration, and white matter injury [9, 34, 47]. In CSF1R-RD, dysfunction in microglial homeostasis due to CSF1R deficiency results in inadequate clearance of myelin debris and other macromolecules, which, exacerbated by chronic inflammation, leads to white matter degeneration [44]. Our snRNAseq analysis revealed two CSF1R-RD-associated activated microglia cell states, M2 and M3, exhibiting gene signatures of pro-inflammation and autophagy, respectively. Notably, our trajectory analysis revealed cell transitions from homeostatic microglia to activated states M2 and M3, with M3 representing an end-stage disease cell state. M3 cells showed high expression of *GPNMB* and other genes involved in lipid metabolism and autophagy (i.e., *CPM, MGLL, PTPRG, SQSTM1, PPARG, HEXA, PLPP3, HS3ST2, LGALS1, LGALS3, SCARB2, ABHD3*). GPNMB has been shown to label lipid-laden macrophages or “foamy cells” in various conditions, including lysosomal storage disorders, cerebral adrenoleukodystrophy, and acquired lipid overload conditions such as Gaucher Disease, Niemann-Pick type C, multiple sclerosis, obesity, and cardiovascular disease [4, 73, 76]. GPNMB has also been described in amyloid plaque-associated microglia in Alzheimer’s disease [25, 66] and recently identified as one the top up-regulated proteins in response to progranulin deficiency [23]. Our immunohistochemistry data using an antibody against GPNMB demonstrated abundant rounded cells with few or no processes, particularly enriched in areas of white matter degeneration. Thus, our M3 cluster and GPNMB^+^ cells likely represent the so-called “pigmented glia” of CSF1R-RD and share molecular signatures with other conditions featuring lipid-laden macrophages. The lipid-droplet-accumulating microglia reported in the aging brain may represent part of the spectrum of lipid accumulation in microglia, although these cells were not reported to express high levels of GPNMB or autophagy signatures [43].

Cluster M4 within the microglia population was small but notably represented infiltrating monocyte-derived macrophages, identified by their distinct expression of *CD44, CCDC88C, MARCO, F13A1*, and *ITGA4* [5, 22, 31, 45, 70]. Further investigation comparing microglia and monocyte-derived macrophages in CSF1R-RD is warranted. This research is pertinent as hematopoietic stem cell transplantation (HSCT) has recently emerged as a therapeutic option for CSF1R-RD patients [3, 12, 20, 74]. Despite the typical impermeability of the blood–brain barrier to peripheral blood cells in healthy brains, during HSCT these cells gradually engraft into the donor brain, differentiating into parenchymal macrophages [65]. Notably, they may assume functional roles akin to resident microglia, potentially offering therapeutic benefits. Given our findings that suggest CSF1R changes precede white matter degeneration, the timing of the transplant may be crucial, with the greatest therapeutic benefit likely achieved if performed in the early stages of the disease.

Our snRNAseq analysis contributes to understanding microglia–oligodendroglia crosstalk in demyelination. The depletion of myelinating oligodendrocytes is a key feature of CSF1R-RD. Our analysis revealed an CSF1R-RD-associated oligodendroglia state (cluster O3) characterized by signatures of cell stress, cell death, and inflammation, as evidenced by the upregulation of *DNAJB1, DNAJB6, HSP90AA1, HSP90AB1, HSPA1A, CRYAB, FOS,* and *TNFRSF12A*. Additionally, we identified an arrest in OPC differentiation, as evidenced by the dysregulation of transcription factors and other genes critical for OPC differentiation (upregulation of *LRP1, PDGFRA, SOX5, SOX6,* and *NFIA*; downregulation of *NKX2-2, NKX6.2, SOX4, SOX8, TCF7L2, YY1,* and *ZNF488)* and downregulation of marker genes of mature oligodendrocytes (*MBP, MOG, S100B, ITGB1*) in late-stage OPCs. The arrest of OPC differentiation has also been demonstrated in demyelinating disorders such as MS and neurodegeneration [4, 24, 29, 49], suggesting a potentially universal mechanism aimed at preventing a vicious neuroinflammatory cycle triggered by myelin debris [39].

## Supplementary Information

Additional file 1 (XLSX 2663 KB). **Supplementary Table 1**. Lists of marker genes for each astrocyte, oligodendrocyte, OPC, and microglia cell state. For each major glial cell type, nuclei derived from each cluster representing a state were compared to nuclei derived from all other clusters using Wilcoxon test analysis.

Additional file 2 (XLSX 6148 KB). **Supplementary Table 2**. Lists of differentially expressed genes (MAST test) for the following comparisons: (1) A1-CSF1R-RD versus A0-Control, (2) A2-CSF1R-RD versus A0-Control, (3) M0-AD versus M1-Control, (4) M2-CSF1R-RD versus M1-Control, (4) M3-CSF1R-RD versus M1-Control, (5) M3-CSF1R-RD versus M2-CSF1R-RD, (6) O3-CSF1R-RD versus O1-Control, (7) OP0-CSF1R-RD versus OP0-Control, (8) OP1-CSF1R-RD versus OP0-Control, (9) OP1-CSF1R-RD versus OP2 + OP3-Control.

Additional file 3 (XLSX 10033 KB). **Supplementary Table 3**. Gene Set Enrichment Analysis results employing the Biological Process branch of Gene Ontology for the following comparisons: (1) A1-CSF1R-RD versus A0-Control, (2) A2-CSF1R-RD versus A0-Control, (3) M0-AD versus M1-Control, (4) M2-CSF1R-RD versus M1-Control, (4) M3-CSF1R-RD versus M1-Control, (5) M3-CSF1R-RD versus M2-CSF1R-RD, (6) O3-CSF1R-RD versus O1-Control, (7) OP0-CSF1R-RD versus OP0-Control, (8) OP1-CSF1R-RD versus OP0-Control, (9) OP1-CSF1R-RD versus OP2 + OP3-Control.

Additional file 4 (XLSX 141 KB). **Supplementary Table 4**. Gene Set Enrichment Analysis results employing the Reactome for the following comparisons: (1) M2-CSF1R-RD versus M1-Control, (2) M3-CSF1R-RD versus M1-Control.

Additional file 5 (PDF 11357 KB). **Supplementary Fig. 1**. UMAP plots showing selected top marker genes for each of the five microglia cell states, including phagocytic AD-associated (**a**), homeostatic (**b**), pro-inflammatory CSF1R-RD-associated (**c**), autophagy CSF1R-RD-associated (**d**), and peripheral monocyte-derived macrophages (**e**). **Supplementary Fig. 2.** *CSF1R* expression levels in microglia clusters. **a** UMAPs showing the distribution of CSF1R-RD, healthy, and AD nuclei. **b **Dot plot and violin plot displaying the gene expression levels of *CSF1R* in CSF1R-RD, healthy, and AD groups. **c **UMAP, dot plot and violin plot depicting the gene expression levels of *CSF1R* in microglia clusters. **d** Dot plot and UMAP depicting the gene expression levels of *TREM2* in microglia clusters*.* **e **Dot plot and violin plot showing the gene expression levels of *CSF1R* in samples. *FDR < 0.05, **FDR < 0.01, ****FDR < 0.0001 (FDR-adjusted p-values using MAST). **Supplementary Fig. 3**. GPNMB and Iba1 immunohistochemistry in frontal cortex (**a**–**d**), anterior cingulate **(e**–**h**), and cerebellum (**i**–**l**; ml: molecular layer; gl: granular cell layer) in CSF1R-RD. Representative images of frontal cortex from a healthy donor (**m**,**n**) demonstrates Iba1^+^ ramified microglia. Dashed lines indicate borders between gray matter (gm) and while matter (wm). Scale bars: 200 µm (**a, c, e, g, i, k, m**); 50 µm (**b, d, f, h, j, I, n**). **Supplementary Fig. 4**. snRNAseq of astrocyte cell states in CSF1R-RD. **a** UMAP plots showing the contributions from CSF1R-RD, healthy control, and AD (left), and from each individual sample (right) to the dataset. **b** UMAP and bar plots showing the four annotated astrocyte states (A0–A3). **c** Contribution of each astrocyte state per sample. **d** Contribution of each disease group to each astrocyte state cluster. **e** Dot plot showing top astrocyte cell state marker genes per cluster (A0: homeostatic; A1–3: reactive). **Supplementary Fig. 5**. UMAP plots showing selected top marker genes for each of the three oligodendroglia cell states (O1–O3; **a**–**c**).

## Data Availability

The raw snRNAseq data generated in this study, associated metadata, and processed digital expression matrices have been deposited at the NCBI's Gene Expression Omnibus with accession number GSE267301. The datasets are publicly available for interactive viewing and exploration on the cellxgene platform at https://cellxgene.cziscience.com/collections/7c4552fd-8a6d-4da3-9854-2dfa8baca8bf.

## References

[1] Berdowski WM, van der Linde HC, Breur M, Oosterhof N, Beerepoot S, Sanderson L, Wijnands LI, de Jong P, Tsai-Meu-Chong E, de Valk W et al (2022) Dominant-acting CSF1R variants cause microglial depletion and altered astrocytic phenotype in zebrafish and adult-onset leukodystrophy. Acta Neuropathol 144:211–239. 10.1007/s00401-022-02440-5 PMC9288387 35713703

[2] Bergen V, Lange M, Peidli S, Wolf FA, Theis FJ (2020) Generalizing RNA velocity to transient cell states through dynamical modeling. Nat Biotechnol 38:1408–1414. 10.1038/s41587-020-0591-3 32747759

[3] Bergner CG, Schafer L, Vucinic V, Schetschorke B, Lier J, Scherlach C, Rullmann M, Sabri O, Classen J, Platzbecker U et al (2023) Case report: Treatment of advanced CSF1-receptor associated leukoencephalopathy with hematopoietic stem cell transplant. Front Neurol 14:1163107. 10.3389/fneur.2023.1163107 PMC10246448 37292133

[4] Boven LA, Van Meurs M, Van Zwam M, Wierenga-Wolf A, Hintzen RQ, Boot RG, Aerts JM, Amor S, Nieuwenhuis EE, Laman JD (2006) Myelin-laden macrophages are anti-inflammatory, consistent with foam cells in multiple sclerosis. Brain 129:517–526. 10.1093/brain/awh707 16364958

[5] Bowman RL, Klemm F, Akkari L, Pyonteck SM, Sevenich L, Quail DF, Dhara S, Simpson K, Gardner EE, Iacobuzio-Donahue CA et al (2016) Macrophage ontogeny underlies differences in tumor-specific education in brain malignancies. Cell Rep 17:2445–2459. 10.1016/j.celrep.2016.10.052 PMC5450644 27840052

[6] Chitu V, Biundo F, Shlager GGL, Park ES, Wang P, Gulinello ME, Gokhan S, Ketchum HC, Saha K, DeTure MA et al (2020) Microglial homeostasis requires balanced CSF-1/CSF-2 receptor signaling. Cell Rep 30(3004–3019):e3005. 10.1016/j.celrep.2020.02.028 PMC7370656 32130903

[7] Chitu V, Gokhan S, Gulinello M, Branch CA, Patil M, Basu R, Stoddart C, Mehler MF, Stanley ER (2015) Phenotypic characterization of a Csf1r haploinsufficient mouse model of adult-onset leukodystrophy with axonal spheroids and pigmented glia (ALSP). Neurobiol Dis 74:219–228. 10.1016/j.nbd.2014.12.001 PMC4323933 25497733

[8] Codjia P, Ayrignac X, Mochel F, Mouzat K, Carra-Dalliere C, Castelnovo G, Ellie E, Etcharry-Bouyx F, Verny C, Belliard S et al (2018) Adult-onset leukoencephalopathy with axonal spheroids and pigmented glia: an MRI study of 16 French cases. AJNR Am J Neuroradiol 39:1657–1661. 10.3174/ajnr.A5744 PMC7655300 30115677

[9] Colonna M, Butovsky O (2017) Microglia function in the central nervous system during health and neurodegeneration. Annu Rev Immunol 35:441–468. 10.1146/annurev-immunol-051116-052358 PMC8167938 28226226

[10] Dai DL, Li M, Lee EB (2023) Human Alzheimer’s disease reactive astrocytes exhibit a loss of homeostastic gene expression. Acta Neuropathol Commun 11:127. 10.1186/s40478-023-01624-8 PMC10398957 37533101

[11] Deczkowska A, Weiner A, Amit I (2020) The physiology, pathology, and potential therapeutic applications of the TREM2 signaling pathway. Cell 181:1207–1217. 10.1016/j.cell.2020.05.003 32531244

[12] Dulski J, Heckman MG, White LJ, Zur-Wyrozumska K, Lund TC, Wszolek ZK (2022) Hematopoietic stem cell transplantation in CSF1R-related leukoencephalopathy: retrospective study on predictors of outcomes. Pharmaceutics. 10.3390/pharmaceutics14122778 PMC9788080 36559271

[13] Dulski J, Koga S, Dickson DW, Wszolek ZK (2023) Report of A family with adult-onset leukoencephalopathy with axonal spheroids and pigmented glia (ALSP) without mutations in CSF1R, AARS1 or AARS2. Mov Disord Clin Pract 10:307–312. 10.1002/mdc3.13650 PMC9941916 36825047

[14] Dulski J, Muthusamy K, Lund TC, Wszolek ZK (2024) CSF1R-related disorder: State of the art, challenges, and proposition of a new terminology. Parkinsonism Relat Disord 121:105894. 10.1016/j.parkreldis.2023.105894 37839910

[15] Erblich B, Zhu L, Etgen AM, Dobrenis K, Pollard JW (2011) Absence of colony stimulation factor-1 receptor results in loss of microglia, disrupted brain development and olfactory deficits. PLoS ONE 6:e26317. 10.1371/journal.pone.0026317 PMC3203114 22046273

[16] Escartin C, Galea E, Lakatos A, O’Callaghan JP, Petzold GC, Serrano-Pozo A, Steinhauser C, Volterra A, Carmignoto G, Agarwal A et al (2021) Reactive astrocyte nomenclature, definitions, and future directions. Nat Neurosci 24:312–325. 10.1038/s41593-020-00783-4 PMC8007081 33589835

[17] Ferrer I (2022) The primary microglial leukodystrophies: a review. Int J Mol Sci. 10.3390/ijms23116341 PMC9181167 35683020

[18] Finak G, McDavid A, Yajima M, Deng J, Gersuk V, Shalek AK, Slichter CK, Miller HW, McElrath MJ, Prlic M et al (2015) MAST: a flexible statistical framework for assessing transcriptional changes and characterizing heterogeneity in single-cell RNA sequencing data. Genome Biol 16:278. 10.1186/s13059-015-0844-5 PMC4676162 26653891

[19] Gazestani V, Kamath T, Nadaf NM, Dougalis A, Burris SJ, Rooney B, Junkkari A, Vanderburg C, Pelkonen A, Gomez-Budia M et al (2023) Early Alzheimer’s disease pathology in human cortex involves transient cell states. Cell 186(4438–4453):e4423. 10.1016/j.cell.2023.08.005 PMC11107481 37774681

[20] Gelfand JM, Greenfield AL, Barkovich M, Mendelsohn BA, Van Haren K, Hess CP, Mannis GN (2020) Allogeneic HSCT for adult-onset leukoencephalopathy with spheroids and pigmented glia. Brain 143:503–511. 10.1093/brain/awz390 31840744

[21] Griffiths JA, Richard AC, Bach K, Lun ATL, Marioni JC (2018) Detection and removal of barcode swapping in single-cell RNA-seq data. Nat Commun 9:2667. 10.1038/s41467-018-05083-x PMC6039488 29991676

[22] Haage V, Semtner M, Vidal RO, Hernandez DP, Pong WW, Chen Z, Hambardzumyan D, Magrini V, Ly A, Walker J et al (2019) Comprehensive gene expression meta-analysis identifies signature genes that distinguish microglia from peripheral monocytes/macrophages in health and glioma. Acta Neuropathol Commun 7:20. 10.1186/s40478-019-0665-y PMC6376799 30764877

[23] Huang M, Modeste E, Dammer E, Merino P, Taylor G, Duong DM, Deng Q, Holler CJ, Gearing M, Dickson D et al (2020) Network analysis of the progranulin-deficient mouse brain proteome reveals pathogenic mechanisms shared in human frontotemporal dementia caused by GRN mutations. Acta Neuropathol Commun 8:163. 10.1186/s40478-020-01037-x PMC7541308 33028409

[24] Hume DA, Caruso M, Ferrari-Cestari M, Summers KM, Pridans C, Irvine KM (2020) Phenotypic impacts of CSF1R deficiencies in humans and model organisms. J Leukoc Biol 107:205–219. 10.1002/JLB.MR0519-143R 31330095

[25] Huttenrauch M, Ogorek I, Klafki H, Otto M, Stadelmann C, Weggen S, Wiltfang J, Wirths O (2018) Glycoprotein NMB: a novel Alzheimer’s disease associated marker expressed in a subset of activated microglia. Acta Neuropathol Commun 6:108. 10.1186/s40478-018-0612-3 PMC6194687 30340518

[26] Hyman BT, Phelps CH, Beach TG, Bigio EH, Cairns NJ, Carrillo MC, Dickson DW, Duyckaerts C, Frosch MP, Masliah E et al (2012) National Institute on Aging-Alzheimer’s Association guidelines for the neuropathologic assessment of Alzheimer’s disease. Alzheimers Dement 8:1–13. 10.1016/j.jalz.2011.10.007 PMC3266529 22265587

[27] Ikeuchi T, Mezaki N, Miura T (2018) Cognitive dysfunction and symptoms of movement disorders in adult-onset leukoencephalopathy with axonal spheroids and pigmented glia. Parkinsonism Relat Disord 46(Suppl 1):S39-s41. 10.1016/j.parkreldis.2017.08.018 28827005

[28] Ishiguro T, Konno T, Hara N, Zhu B, Okada S, Shibata M, Saika R, Kitano T, Toko M, Nezu T et al (2023) Novel partial deletions, frameshift and missense mutations of CSF1R in patents with CSF1R-related leukoencephalopathy. Eur J Neurol 30:1861–1870. 10.1111/ene.15796 36943150

[29] Jain A, Arena VP, Steigerwald C, Borja MJ, Kister I, Abreu NJ (2023) Pearls & Oy-sters: CSF1R-related leukoencephalopathy with spinal cord lesions mimicking multiple sclerosis. Neurology 101:e1178–e1181. 10.1212/wnl.0000000000207502 PMC10513882 37407261

[30] Jakel S, Agirre E, Mendanha Falcao A, van Bruggen D, Lee KW, Knuesel I, Malhotra D, Ffrench-Constant C, Williams A, Castelo-Branco G (2019) Altered human oligodendrocyte heterogeneity in multiple sclerosis. Nature 566:543–547. 10.1038/s41586-019-0903-2 PMC6544546 30747918

[31] Jordao MJC, Sankowski R, Brendecke SM, Sagar LG, Tai YH, Tay TL, Schramm E, Armbruster S, Hagemeyer N et al (2019) Single-cell profiling identifies myeloid cell subsets with distinct fates during neuroinflammation. Science. 10.1126/science.aat7554 30679343

[32] Kempthorne L, Yoon H, Madore C, Smith S, Wszolek ZK, Rademakers R, Kim J, Butovsky O, Dickson DW (2020) Loss of homeostatic microglial phenotype in CSF1R-related Leukoencephalopathy. Acta Neuropathol Commun 8:72. 10.1186/s40478-020-00947-0 PMC7236286 32430064

[33] Kenigsbuch M, Bost P, Halevi S, Chang Y, Chen S, Ma Q, Hajbi R, Schwikowski B, Bodenmiller B, Fu H et al (2022) A shared disease-associated oligodendrocyte signature among multiple CNS pathologies. Nat Neurosci 25:876–886. 10.1038/s41593-022-01104-7 PMC9724210 35760863

[34] Kent SA, Miron VE (2024) Microglia regulation of central nervous system myelin health and regeneration. Nat Rev Immunol 24:49–63. 10.1038/s41577-023-00907-4 37452201

[35] Konno T, Kasanuki K, Ikeuchi T, Dickson DW, Wszolek ZK (2018) CSF1R-related leukoencephalopathy: a major player in primary microgliopathies. Neurology 91:1092–1104. 10.1212/WNL.0000000000006642 PMC6329328 30429277

[36] Konno T, Tada M, Tada M, Koyama A, Nozaki H, Harigaya Y, Nishimiya J, Matsunaga A, Yoshikura N, Ishihara K et al (2014) Haploinsufficiency of CSF-1R and clinicopathologic characterization in patients with HDLS. Neurology 82:139–148. 10.1212/WNL.0000000000000046 PMC3937843 24336230

[37] Konno T, Yoshida K, Mizuno T, Kawarai T, Tada M, Nozaki H, Ikeda SI, Nishizawa M, Onodera O, Wszolek ZK et al (2017) Clinical and genetic characterization of adult-onset leukoencephalopathy with axonal spheroids and pigmented glia associated with CSF1R mutation. Eur J Neurol 24:37–45. 10.1111/ene.13125 PMC5215554 27680516

[38] Korsunsky I, Millard N, Fan J, Slowikowski K, Zhang F, Wei K, Baglaenko Y, Brenner M, Loh PR, Raychaudhuri S (2019) Fast, sensitive and accurate integration of single-cell data with Harmony. Nat Methods 16:1289–1296. 10.1038/s41592-019-0619-0 PMC6884693 31740819

[39] Kotter MR, Li WW, Zhao C, Franklin RJ (2006) Myelin impairs CNS remyelination by inhibiting oligodendrocyte precursor cell differentiation. J Neurosci 26:328–332. 10.1523/JNEUROSCI.2615-05.2006 PMC6674302 16399703

[40] Leng C, Lu L, Wang G, Zhang Y, Xu Y, Lin X, Shen N, Xu X, Qun S, Sun M et al (2019) A novel dominant-negative mutation of the CSF1R gene causes adult-onset leukoencephalopathy with axonal spheroids and pigmented glia. Am J Transl Res 11:6093–6101 PMC6789214 31632577

[41] Liddelow SA, Barres BA (2017) Reactive astrocytes: production, function, and therapeutic potential. Immunity 46:957–967. 10.1016/j.immuni.2017.06.006 28636962

[42] Lun ATL, Riesenfeld S, Andrews T, Dao TP, Gomes T, participants in the 1st Human Cell Atlas J, Marioni JC (2019) EmptyDrops: distinguishing cells from empty droplets in droplet-based single-cell RNA sequencing data. Genome Biol 20: 63. 10.1186/s13059-019-1662-y PMC6431044 30902100

[43] Marschallinger J, Iram T, Zardeneta M, Lee SE, Lehallier B, Haney MS, Pluvinage JV, Mathur V, Hahn O, Morgens DW et al (2020) Lipid-droplet-accumulating microglia represent a dysfunctional and proinflammatory state in the aging brain. Nat Neurosci 23:194–208. 10.1038/s41593-019-0566-1 PMC7595134 31959936

[44] Marzan DE, Brugger-Verdon V, West BL, Liddelow S, Samanta J, Salzer JL (2021) Activated microglia drive demyelination via CSF1R signaling. Glia 69:1583–1604. 10.1002/glia.23980 PMC9250806 33620118

[45] Masuda T, Sankowski R, Staszewski O, Bottcher C, Amann L, Sagar SC, Nessler S, Kunz P, van Loo G et al (2019) Spatial and temporal heterogeneity of mouse and human microglia at single-cell resolution. Nature 566:388–392. 10.1038/s41586-019-0924-x 30760929

[46] McGinnis CS, Murrow LM, Gartner ZJ (2019) DoubletFinder: doublet detection in single-cell RNA sequencing data using artificial nearest neighbors. Cell Syst 8(329–337):e324. 10.1016/j.cels.2019.03.003 PMC6853612 30954475

[47] McNamara NB, Munro DAD, Bestard-Cuche N, Uyeda A, Bogie JFJ, Hoffmann A, Holloway RK, Molina-Gonzalez I, Askew KE, Mitchell S et al (2023) Microglia regulate central nervous system myelin growth and integrity. Nature 613:120–129. 10.1038/s41586-022-05534-y PMC9812791 36517604

[48] Miedema A, Gerrits E, Brouwer N, Jiang Q, Kracht L, Meijer M, Nutma E, Peferoen-Baert R, Pijnacker ATE, Wesseling EM et al (2022) Brain macrophages acquire distinct transcriptomes in multiple sclerosis lesions and normal appearing white matter. Acta Neuropathol Commun 10:8. 10.1186/s40478-021-01306-3 PMC8796391 35090578

[49] Misirocchi F, Zilioli A, Benussi A, Capellari S, Mutti C, Florindo I, Spallazzi M, Parrino L (2022) A novel CSF1R mutation mimicking frontotemporal dementia: a glimpse into a microgliopathy - CORRIGENDUM. Can J Neurol Sci. 10.1017/cjn.2022.302 35726564

[50] Muthusamy K, Sivadasan A, Dixon L, Sudhakar S, Thomas M, Danda S, Wszolek ZK, Wierenga K, Dhamija R, Gavrilova R (2023) Adult-onset leukodystrophies: a practical guide, recent treatment updates, and future directions. Front Neurol 14:1219324. 10.3389/fneur.2023.1219324 PMC10410460 37564735

[51] Nandi S, Gokhan S, Dai XM, Wei S, Enikolopov G, Lin H, Mehler MF, Stanley ER (2012) The CSF-1 receptor ligands IL-34 and CSF-1 exhibit distinct developmental brain expression patterns and regulate neural progenitor cell maintenance and maturation. Dev Biol 367:100–113. 10.1016/j.ydbio.2012.03.026 PMC3388946 22542597

[52] Neal ML, Boyle AM, Budge KM, Safadi FF, Richardson JR (2018) The glycoprotein GPNMB attenuates astrocyte inflammatory responses through the CD44 receptor. J Neuroinflammation 15:73. 10.1186/s12974-018-1100-1 PMC5842560 29519253

[53] Nicholson AM, Baker MC, Finch NA, Rutherford NJ, Wider C, Graff-Radford NR, Nelson PT, Clark HB, Wszolek ZK, Dickson DW et al (2013) CSF1R mutations link POLD and HDLS as a single disease entity. Neurology 80:1033–1040. 10.1212/WNL.0b013e31828726a7 PMC3653204 23408870

[54] Oosterhof N, Chang IJ, Karimiani EG, Kuil LE, Jensen DM, Daza R, Young E, Astle L, van der Linde HC, Shivaram GM et al (2019) Homozygous mutations in CSF1R cause a pediatric-onset leukoencephalopathy and can result in congenital absence of microglia. Am J Hum Genet 104:936–947. 10.1016/j.ajhg.2019.03.010 PMC6506793 30982608

[55] Oyanagi K, Kinoshita M, Suzuki-Kouyama E, Inoue T, Nakahara A, Tokiwai M, Arai N, Satoh JI, Aoki N, Jinnai K et al (2017) Adult onset leukoencephalopathy with axonal spheroids and pigmented glia (ALSP) and Nasu-Hakola disease: lesion staging and dynamic changes of axons and microglial subsets. Brain Pathol 27:748–769. 10.1111/bpa.12443 PMC8029200 27608278

[56] Pandey S, Shen K, Lee SH, Shen YA, Wang Y, Otero-Garcia M, Kotova N, Vito ST, Laufer BI, Newton DF et al (2022) Disease-associated oligodendrocyte responses across neurodegenerative diseases. Cell Rep 40:111189. 10.1016/j.celrep.2022.111189 36001972

[57] Paolicelli RC, Sierra A, Stevens B, Tremblay ME, Aguzzi A, Ajami B, Amit I, Audinat E, Bechmann I, Bennett M et al (2022) Microglia states and nomenclature: A field at its crossroads. Neuron 110:3458–3483. 10.1016/j.neuron.2022.10.020 PMC9999291 36327895

[58] Papapetropoulos S, Gelfand JM, Konno T, Ikeuchi T, Pontius A, Meier A, Foroutan F, Wszolek ZK (2024) Clinical presentation and diagnosis of adult-onset leukoencephalopathy with axonal spheroids and pigmented glia: a literature analysis of case studies. Front Neurol 15:1320663. 10.3389/fneur.2024.1320663 PMC10962389 38529036

[59] Papapetropoulos S, Pontius A, Finger E, Karrenbauer V, Lynch DS, Brennan M, Zappia S, Koehler W, Schoels L, Hayer SN et al (2021) Adult-onset leukoencephalopathy with axonal spheroids and pigmented glia: review of clinical manifestations as foundations for therapeutic development. Front Neurol 12:788168. 10.3389/fneur.2021.788168 PMC8850408 35185751

[60] Pridans C, Sauter KA, Baer K, Kissel H, Hume DA (2013) CSF1R mutations in hereditary diffuse leukoencephalopathy with spheroids are loss of function. Sci Rep 3:3013. 10.1038/srep03013 PMC3804858 24145216

[61] Prieto-Morin C, Ayrignac X, Ellie E, Tournier-Lasserve E, Labauge P (2016) CSF1R-related leukoencephalopathy mimicking primary progressive multiple sclerosis. J Neurol 263:1864–1865. 10.1007/s00415-016-8197-x 27314966

[62] Rademakers R, Baker M, Nicholson AM, Rutherford NJ, Finch N, Soto-Ortolaza A, Lash J, Wider C, Wojtas A, DeJesus-Hernandez M et al (2011) Mutations in the colony stimulating factor 1 receptor (CSF1R) gene cause hereditary diffuse leukoencephalopathy with spheroids. Nat Genet 44:200–205. 10.1038/ng.1027 PMC3267847 22197934

[63] Riku Y, Ando T, Goto Y, Mano K, Iwasaki Y, Sobue G, Yoshida M (2014) Early pathologic changes in hereditary diffuse leukoencephalopathy with spheroids. J Neuropathol Exp Neurol 73:1183–1190. 10.1097/NEN.0000000000000139 25383640

[64] Sadick JS, O’Dea MR, Hasel P, Dykstra T, Faustin A, Liddelow SA (2022) Astrocytes and oligodendrocytes undergo subtype-specific transcriptional changes in Alzheimer’s disease. Neuron 110(1788–1805):e1710. 10.1016/j.neuron.2022.03.008 PMC9167747 35381189

[65] Sailor KA, Agoranos G, Lopez-Manzaneda S, Tada S, Gillet-Legrand B, Guerinot C, Masson JB, Vestergaard CL, Bonner M, Gagnidze K et al (2022) Hematopoietic stem cell transplantation chemotherapy causes microglia senescence and peripheral macrophage engraftment in the brain. Nat Med 28:517–527. 10.1038/s41591-022-01691-9 35190726

[66] Satoh JI, Kino Y, Yanaizu M, Ishida T, Saito Y (2019) Microglia express GPNMB in the brains of Alzheimer’s disease and Nasu-Hakola disease. Intractable Rare Dis Res 8:120–128. 10.5582/irdr.2019.01049 PMC6557242 31218162

[67] Schirmer L, Velmeshev D, Holmqvist S, Kaufmann M, Werneburg S, Jung D, Vistnes S, Stockley JH, Young A, Steindel M et al (2019) Neuronal vulnerability and multilineage diversity in multiple sclerosis. Nature 573:75–82. 10.1038/s41586-019-1404-z PMC6731122 31316211

[68] Sharma R, Graff-Radford J, Rademakers R, Boeve BF, Petersen RC, Jones DT (2019) CSF1R mutation presenting as dementia with Lewy bodies. Neurocase 25:17–20. 10.1080/13554794.2019.1601230 30968732

[69] Shetty D, Desai A, Gupta V, Agarwal A (2023) CSF1R-related leukoencephalopathy. J Clin Neurosci 113:60–61. 10.1016/j.jocn.2023.05.008 37209513

[70] Sun N, Victor MB, Park YP, Xiong X, Scannail AN, Leary N, Prosper S, Viswanathan S, Luna X, Boix CA et al (2023) Human microglial state dynamics in Alzheimer’s disease progression. Cell 186(4386–4403):e4329. 10.1016/j.cell.2023.08.037 PMC10644954 37774678

[71] Sundal C, Fujioka S, Van Gerpen JA, Wider C, Nicholson AM, Baker M, Shuster EA, Aasly J, Spina S, Ghetti B et al (2013) Parkinsonian features in hereditary diffuse leukoencephalopathy with spheroids (HDLS) and CSF1R mutations. Parkinsonism Relat Disord 19:869–877. 10.1016/j.parkreldis.2013.05.013 PMC3977389 23787135

[72] Sundal C, Van Gerpen JA, Nicholson AM, Wider C, Shuster EA, Aasly J, Spina S, Ghetti B, Roeber S, Garbern J et al (2012) MRI characteristics and scoring in HDLS due to CSF1R gene mutations. Neurology 79:566–574. 10.1212/WNL.0b013e318263575a PMC3413763 22843259

[73] Taghizadeh LA, King CJ, Nascene DR, Gupta AO, Orchard PJ, Higgins L, Markowski TW, Nolan EE, Furcich JW, Lund TC (2022) Glycoprotein nonmetastatic melanoma protein B (GNMPB) as a novel biomarker for cerebral adrenoleukodystrophy. Sci Rep 12:7985. 10.1038/s41598-022-11552-7 PMC9107455 35568699

[74] Tipton PW, Kenney-Jung D, Rush BK, Middlebrooks EH, Nascene D, Singh B, Holtan S, Ayala E, Broderick DF, Lund T et al (2021) Treatment of CSF1R-related leukoencephalopathy: breaking new ground. Mov Disord 36:2901–2909. 10.1002/mds.28734 34329526

[75] van der Knaap MS, Bugiani M (2017) Leukodystrophies: a proposed classification system based on pathological changes and pathogenetic mechanisms. Acta Neuropathol 134:351–382. 10.1007/s00401-017-1739-1 PMC5563342 28638987

[76] van Eijk M, Aerts J (2021) The unique phenotype of lipid-laden macrophages. Int J Mol Sci. 10.3390/ijms22084039 PMC8070766 33919858

[77] Wade C, Runeckles K, Chataway J, Houlden H, Lynch DS (2024) CSF1R-related disorder: prevalence of CSF1R variants and their clinical significance in the UK population. Neurol Genet 10:e200179. 10.1212/NXG.0000000000200179 PMC11261581 39040919

[78] Wolf FA, Angerer P, Theis FJ (2018) SCANPY: large-scale single-cell gene expression data analysis. Genome Biol 19:15. 10.1186/s13059-017-1382-0 PMC5802054 29409532

[79] Wolf FA, Hamey FK, Plass M, Solana J, Dahlin JS, Gottgens B, Rajewsky N, Simon L, Theis FJ (2019) PAGA: graph abstraction reconciles clustering with trajectory inference through a topology preserving map of single cells. Genome Biol 20:59. 10.1186/s13059-019-1663-x PMC6425583 30890159

[80] Wu J, Cheng X, Ji D, Niu H, Yao S, Lv X, Wang J, Li Z, Zheng H, Cao Y et al (2024) The phenotypic and genotypic spectrum of CSF1R-related disorder in China. Mov Disord 39:798–813. 10.1002/mds.29764 38465843

[81] Zhu Z, Liu Y, Li X, Zhang L, Liu H, Cui Y, Wang Y, Zhao D (2022) GPNMB mitigates Alzheimer’s disease and enhances autophagy via suppressing the mTOR signal. Neurosci Lett 767:136300. 10.1016/j.neulet.2021.136300 34695452

[82] Zhuang LP, Liu CY, Li YX, Huang HP, Zou ZY (2020) Clinical features and genetic characteristics of hereditary diffuse leukoencephalopathy with spheroids due to CSF1R mutation: a case report and literature review. Ann Transl Med 8:11. 10.21037/atm.2019.12.17 PMC6995741 32055602

