# Correction to: Deciphering glial contributions to CSF1R-related disorder via single-nuclear transcriptomic profiling: a case study

**DOI:** 10.1186/s40478-026-02443-3

**Published:** 2026-10-06

**Authors:** Jie Pan, Jaume Fores-Martos, Claire Delpirou Nouh, Tanner D. Jensen, Kristen Vallejo, Romain Cayrol, Saman Ahmadian, Euan A. Ashley, Michael D. Greicius, Inma Cobos

**Affiliations:** 1 Department of Pathology, Stanford University School of Medicine, Stanford, CA USA; 2 Department of Neurology and Neurological Sciences, Stanford University School of Medicine, Stanford, CA USA; 3 Department of Pathology and Cell Biology, Faculty of Medicine, Université de Montréal, Montreal, QC Canada; 4 Department of Pathology, The Ohio State University Wexner Medical Center, Columbus, OH USA; 5 Department of Medicine, Stanford University School of Medicine, Stanford, CA USA; 6 Department of Genetics, Stanford University School of Medicine, Stanford, CA USA

**Correction to: Acta Neuropathologica Communications (2024) 12:139.**

**https://doi.org/10.1186/s40478-024-01853-5**.

In the original publication of this article [1], there was a duplication between Fig. 2c and e in Fig. 2. This has now been corrected. For completeness and transparency, the previous versions are provided below.

The following updates have been made to the original article:

- Page 11: Updated the total number of profiled nuclei from 58,347 to 58,447. The revised sentence reads: “After quality control, we obtained 58,447 high-quality nuclei (17,063 ALPS, 10,348 AD, 31,036 healthy).”
- Page 5: Updated the sentence regarding Scanpy to: “We used Scanpy in the Python package [78], version 3.9.1 to analyze raw count data.”
- Page 5: Corrected the threshold condition for droplet removal to state that droplets with “FDR > 0.05 were removed” instead of “< 0.05”.

Incorrect Fig. 2.

**Fig. 2:**
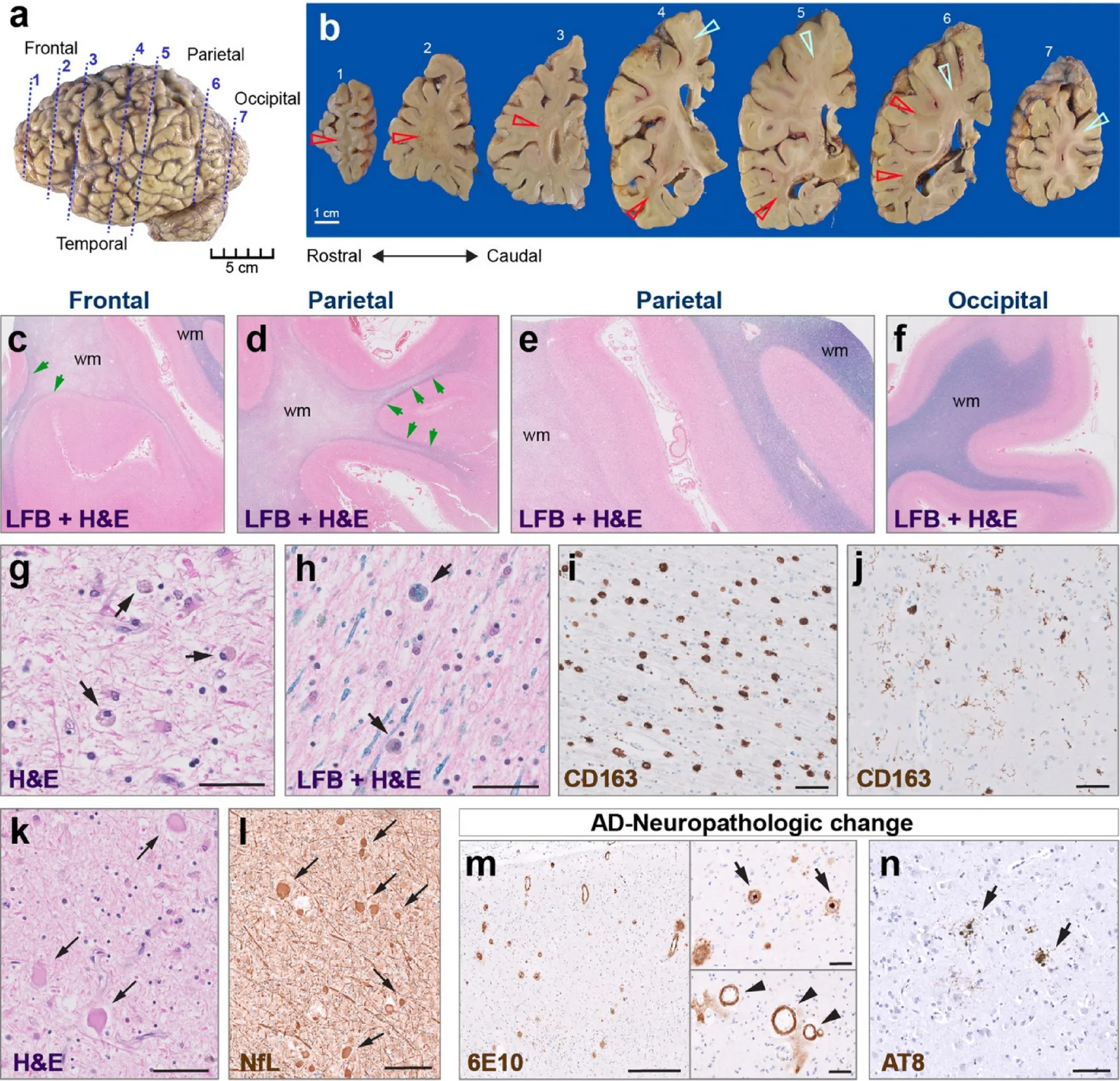
Neuropathology findings of CSF1R-RD and concurrent AD. Lateral view (a) of the formalin-fixed hemibrain (1355 g) shows mild to moderate diffuse cortical atrophy, greater in the frontal lobe. Coronal sections (b) of the left hemisphere demonstrates atrophic white matter with tan-brown discoloration involving the frontal, parietal, and temporal lobes (red arrows) and relatively preserved white matter in the pre- and post-central gyri and occipital lobes (blue arrows). Hematoxylin and eosin (H&E) and Luxol Fast Blue (LFB) stains from FFPE coronal sections through frontal (c), parietal (d, e), and primary visual (f) cortices highlight pale white matter (wm) with loss of myelin and adjacent relatively intact white matter. U fibers (green arrows) are relatively spared. White matter contains pigmented glia (myelin-laden microglia/macrophages; arrows in H&E and LFB + H&E stains) (g, h). CD163 immunostain in sections from white (i) and gray (j) matter highlights ameboid and hyper-ramified/bushy microglia, respectively. H&E and NfL immunostain demonstrate axonal spheroids (k, l; arrows). Amyloid and tau pathology (6E10 and AT8 immunostains) in parietal cortex (m, n) highlight neuritic (arrows) and diffuse plaques, p-tau threads, and cerebral amyloid angiopathy (arrowheads). Scale bars: 50 μm (c–l, n); 500 μm (m)

Correct Fig. 2.

**Fig. 2:**
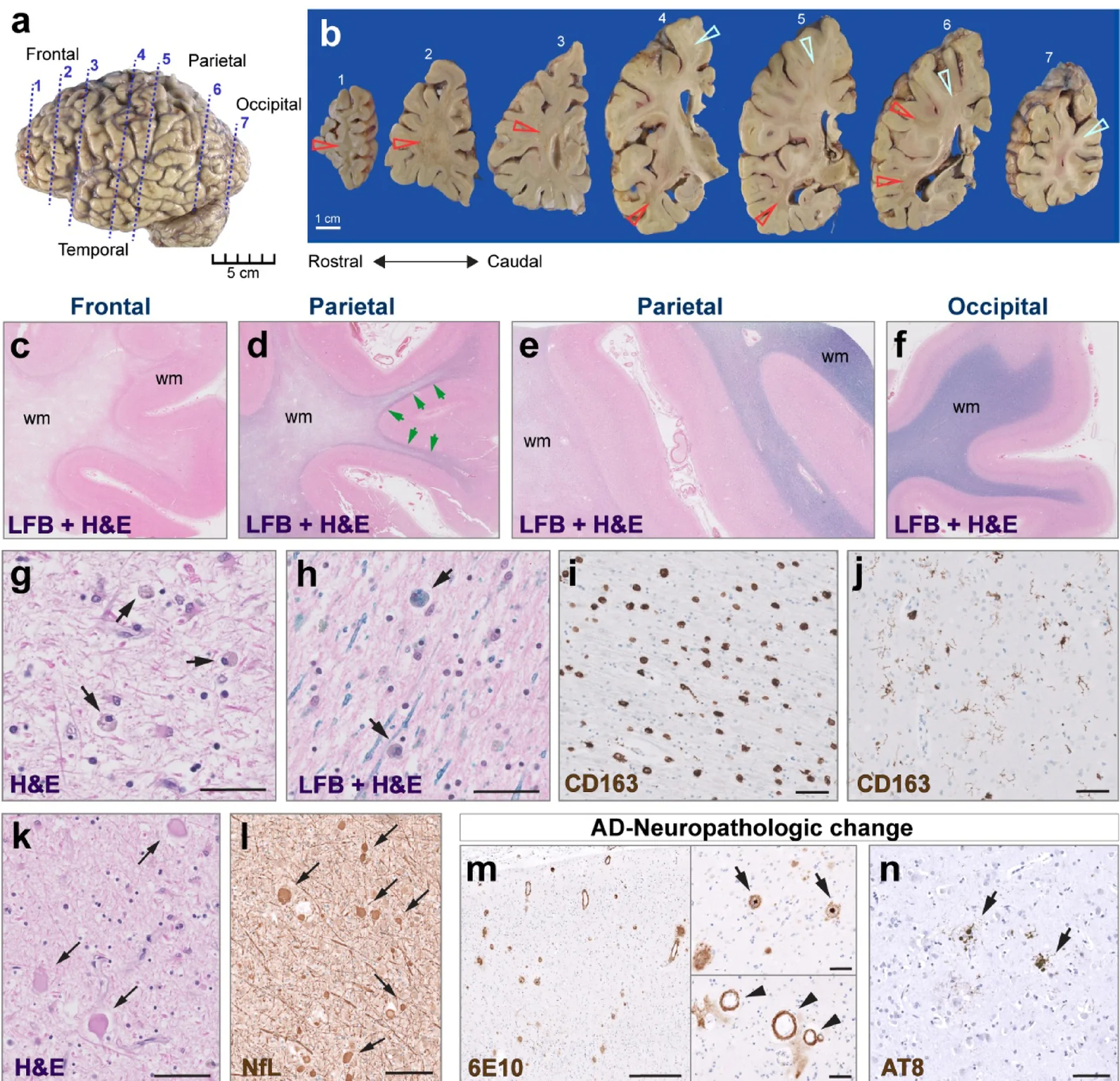
Neuropathology findings of CSF1R-RD and concurrent AD. Lateral view (a) of the formalin-fixed hemibrain (1355 g) shows mild to moderate diffuse cortical atrophy, greater in the frontal lobe. Coronal sections (b) of the left hemisphere demonstrates atrophic white matter with tan-brown discoloration involving the frontal, parietal, and temporal lobes (red arrows) and relatively preserved white matter in the pre- and post-central gyri and occipital lobes (blue arrows). Hematoxylin and eosin (H&E) and Luxol Fast Blue (LFB) stains from FFPE coronal sections through frontal (c), parietal (d, e), and primary visual (f) cortices highlight pale white matter (wm) with loss of myelin and adjacent relatively intact white matter. U fibers (green arrows) are relatively spared. White matter contains pigmented glia (myelin-laden microglia/macrophages; arrows in H&E and LFB + H&E stains) (g, h). CD163 immunostain in sections from white (i) and gray (j) matter highlights ameboid and hyper-ramified/bushy microglia, respectively. H&E and NfL immunostain demonstrate axonal spheroids (k, l; arrows). Amyloid and tau pathology (6E10 and AT8 immunostains) in parietal cortex (m, n) highlight neuritic (arrows) and diffuse plaques, p-tau threads, and cerebral amyloid angiopathy (arrowheads). Scale bars: 50 μm (c–l, n); 500 μm (m)
