# Supplementary figures and images for: Deciphering glial contributions to CSF1R-related disorder via single-nuclear transcriptomic profiling: a case study
Source: Acta Neuropathol Commun. 2024 Aug 28;12:139. doi: 10.1186/s40478-024-01853-5 (PMC11365264; doi:10.1186/s40478-024-01853-5)

**a**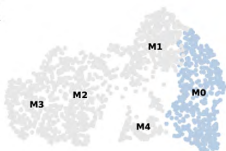**b**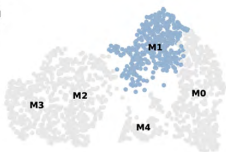**c**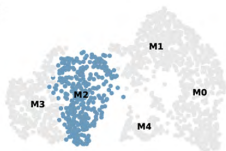**d**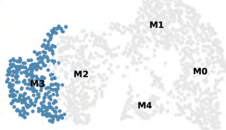**e**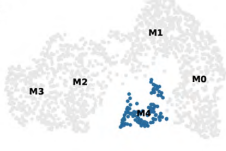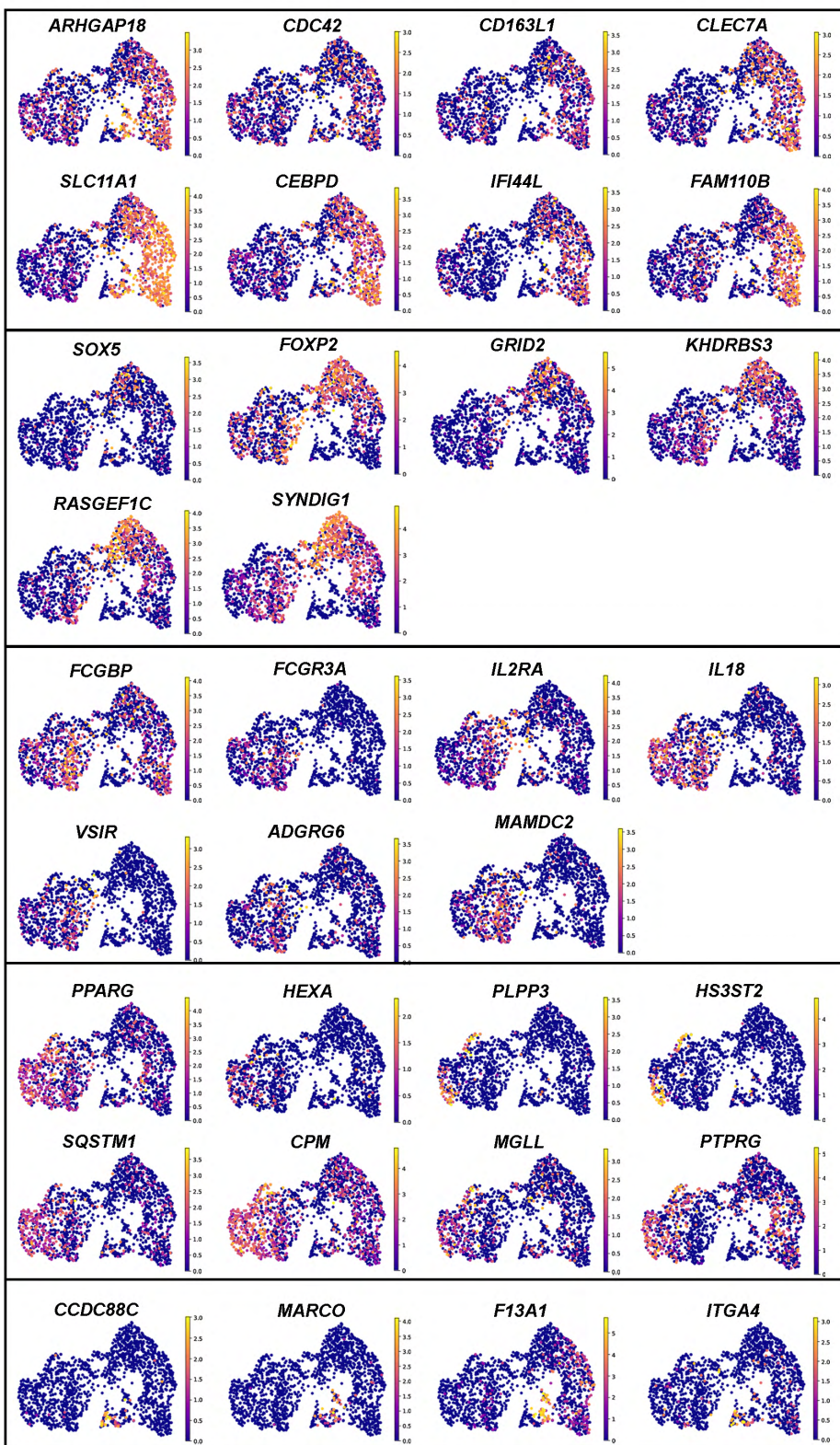

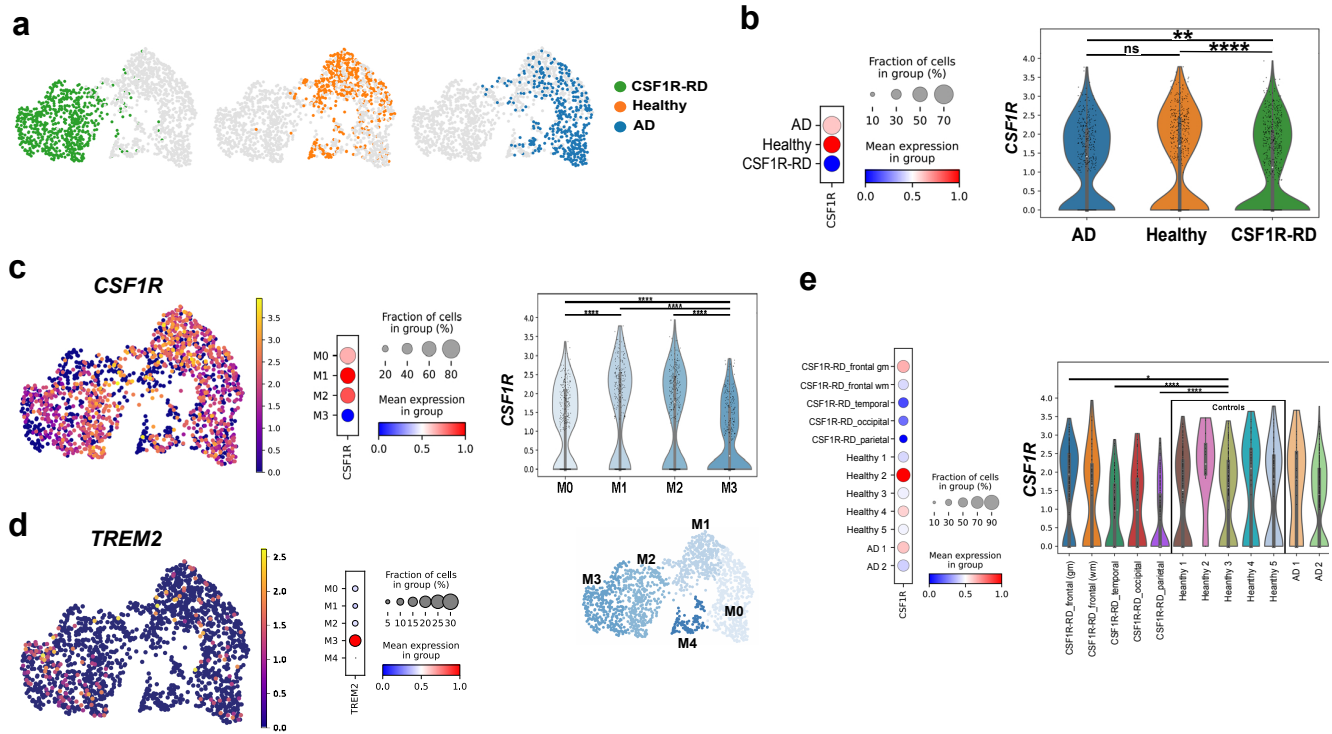

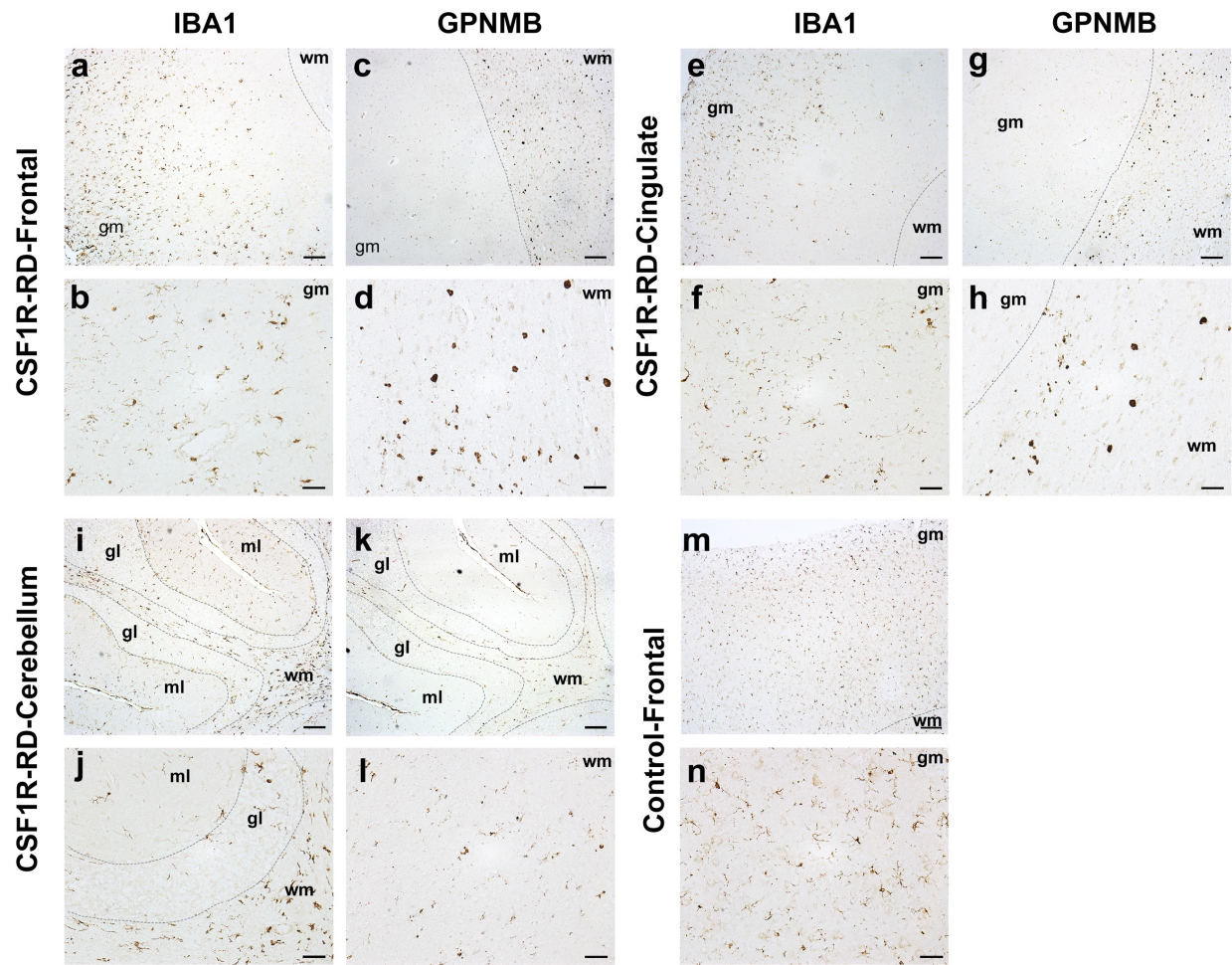

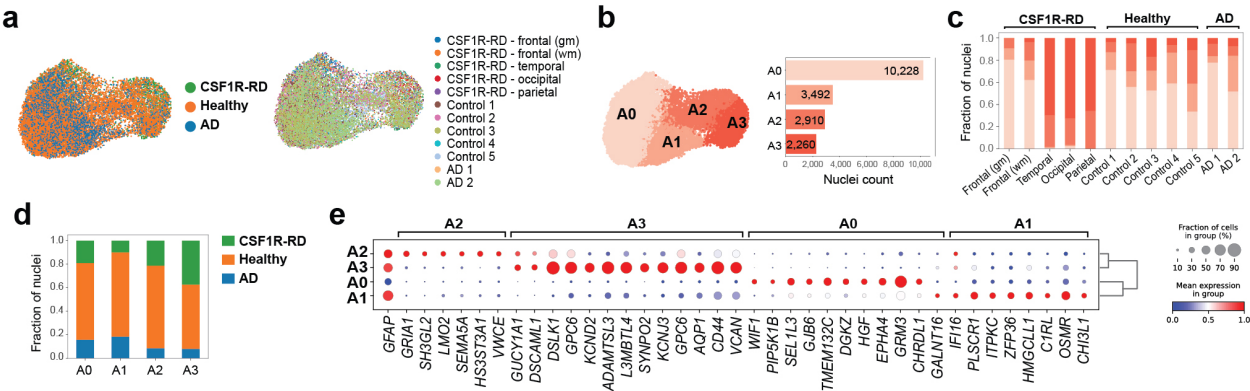

**a**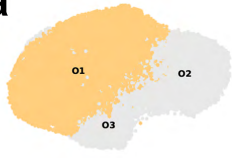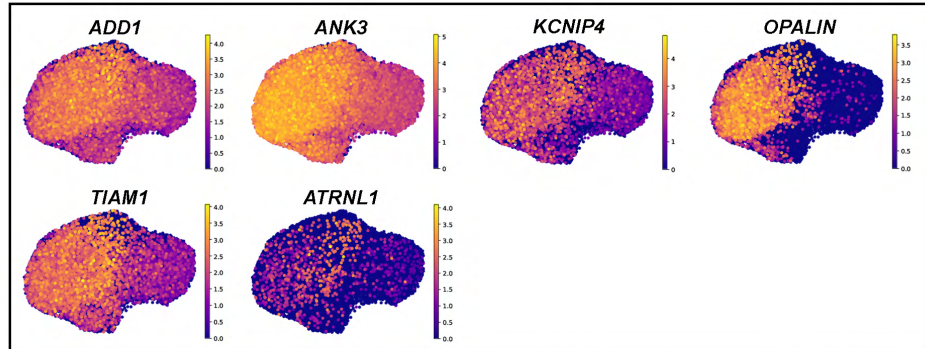**b**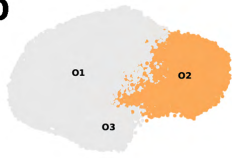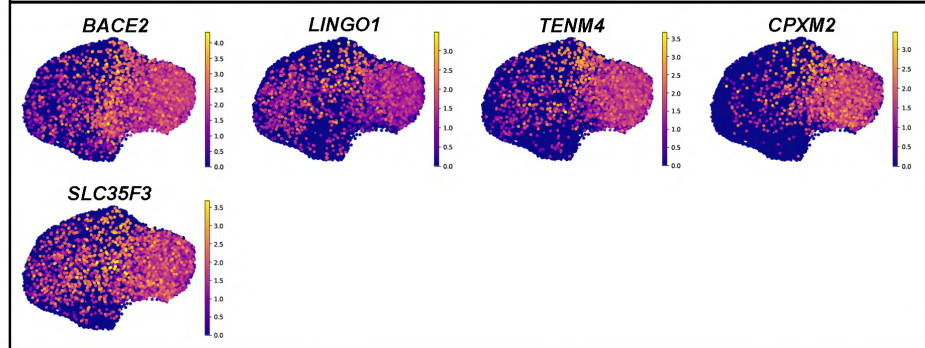**c**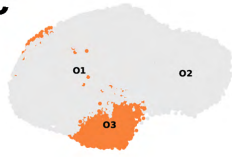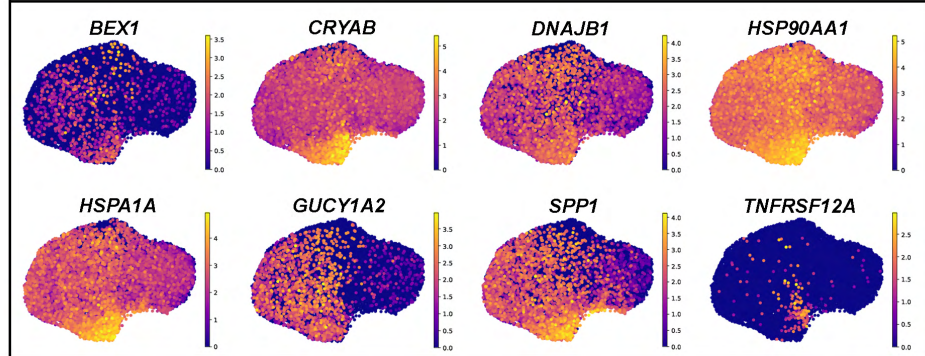

Supplement: Supplementary file 5 — Additional file 5 (PDF 11357 KB). Supplementary Fig. 1. UMAP plots showing selected top marker genes for each of the five microglia cell states, including phagocytic AD-associated (a), homeostatic (b), pro-inflammatory CSF1R-RD-associated (c), autophagy CSF1R-RD-associated (d), and peripheral monocyte-derived macrophages (e). Supplementary Fig. 2. CSF1R expression levels in microglia clusters. a UMAPs showing the distribution of CSF1R-RD, healthy, and AD nuclei. b Dot plot and violin plot displaying the gene expression levels of CSF1R in CSF1R-RD, healthy, and AD groups. c UMAP, dot plot and violin plot depicting the gene expression levels of CSF1R in microglia clusters. d Dot plot and UMAP depicting the gene expression levels of TREM2 in microglia clusters. e Dot plot and violin plot showing the gene expression levels of CSF1R in samples. *FDR < 0.05, **FDR < 0.01, ****FDR < 0.0001 (FDR-adjusted p-values using MAST). Supplementary Fig. 3. GPNMB and Iba1 immunohistochemistry in frontal cortex (a–d), anterior cingulate (e–h), and cerebellum (i–l; ml: molecular layer; gl: granular cell layer) in CSF1R-RD. Representative images of frontal cortex from a healthy donor (m,n) demonstrates Iba1+ ramified microglia. Dashed lines indicate borders between gray matter (gm) and while matter (wm). Scale bars: 200 µm (a, c, e, g, i, k, m); 50 µm (b, d, f, h, j, I, n). Supplementary Fig. 4. snRNAseq of astrocyte cell states in CSF1R-RD. a UMAP plots showing the contributions from CSF1R-RD, healthy control, and AD (left), and from each individual sample (right) to the dataset. b UMAP and bar plots showing the four annotated astrocyte states (A0–A3). c Contribution of each astrocyte state per sample. d Contribution of each disease group to each astrocyte state cluster. e Dot plot showing top astrocyte cell state marker genes per cluster (A0: homeostatic; A1–3: reactive). Supplementary Fig. 5. UMAP plots showing selected top marker genes for each of the three oligodendroglia cell stat [file 40478_2024_1853_MOESM5_ESM.pdf]
